# Mitochondrial and ER stress crosstalk in TBI: mechanistic insights and therapeutic opportunities

**DOI:** 10.3389/fncel.2025.1697060

**Published:** 2025-12-17

**Authors:** Luo Wenzhe, Xia Boyang, Gong Yuchao, Riji Bimcle, Yin Yue

**Affiliations:** School of Basic Medicine, Jiamusi University, Jiamusi, China

**Keywords:** apoptosis and autophagy, calcium signaling, MAMS, neuroinflammation, oxidative and ER stress, TBI

## Abstract

Traumatic brain injury (TBI) remains a major global public health concern, characterized by high morbidity, mortality, and long-term disability. Beyond the primary mechanical insult, the progression of secondary injuries—including neuroinflammation, oxidative stress, mitochondrial dysfunction, and excitotoxicity—plays a decisive role in long-term neurological outcomes. Emerging evidence positions cellular stress responses at the core of TBI pathophysiology, mediating the transition from acute injury to chronic neurodegeneration. This review systematically outlines the major stress phenotypes triggered by TBI, including oxidative stress, endoplasmic reticulum (ER) stress, mitochondrial distress, and autophagy imbalance. Particular emphasis is placed on the molecular interplay between the mitochondria and ER, where the mitochondria-associated membranes (MAMs) serve as dynamic hubs regulating calcium (Ca^2+^) homeostasis, ATP production, and apoptotic signaling. Disruptions in Ca^2+^ flux through MAMs exacerbate energy failure and promote reactive oxygen species (ROS) overproduction, triggering pro-inflammatory cascades and neuronal apoptosis. Furthermore, the crosstalk between ER-mitochondrial stress integrates signals that govern autophagy and inflammatory responses via key nodes such as C/EBP Homologous Protein (CHOP), Nuclear factor erythroid 2–related factor 2(Nrf2), and Nuclear Factor kappa-light-chain-enhancer of activated B cells (NF-κB). We also explore how stress crosstalk mechanistically contributes to neurological dysfunctions, including glial activation, axonal injury, and progressive cognitive-behavioral impairments. Understanding these intricate molecular mechanisms not only elucidates the pathogenesis of secondary brain damage but also unveils novel therapeutic targets for intervention. Targeting stress response integration may represent a transformative approach in preventing long-term disability and enhancing neuroregenerative outcomes following TBI.

## 1 Introduction

Traumatic brain injury (TBI) continues to represent a major global health concern, contributing to substantial mortality and long-term disability across (Liu J)diverse populations. Recent data from the Global Burden of Disease Study estimate that nearly 50 million individuals suffer new TBI episodes annually, with the most frequent causes including road traffic incidents, falls, and violent encounters (Liu et al., 2025). While improvements in acute medical interventions and neurosurgical protocols have enhanced early survival, the overall burden of TBI remains disproportionately high—particularly in low- and middle-income settings (Gu et al., 2025). Clinically, TBI is characterized by extreme heterogeneity in presentation and course and is determined by the level of damage, the anatomical location, and local cellular reactions (McDonald et al., 2024). This is very complex and poses constant problems during diagnosis, prognostication and management. Moreover, secondary complications with the increase of intracranial pressure, the risk of seizure, and chronic neuroinflammation are strongly connected with unsuccessful neurological outcomes and long-lasting cognitive alteration (van Erp et al., 2023).

The first mechanical insult, or what may be considered as the primary injury, induces a sequential cascade of secondary pathophysiological events. These include excitotoxic neurotransmitter release, accumulation of reactive oxygen species (ROS), mitochondrial dysfunction, and the activation of glia related to inflammatory processes causing cell damage beyond the initial focal point of damage (de Macedo Filho et al., 2024). Treatment during this delayed period is one of the main neuroprotection processes because in-time therapeutic modification can prevent apoptosis of neurons and increase recovery over time (Bahuguna et al., 2025). More importantly, the elucidation of the complex molecular networks governing this changing pathology is at the center of unveiling access point therapeutic avenues. The cellular and systemic stress responses are some of these networks that are becoming more and more popular. Post-injury activation of the hypothalamic–pituitary–adrenal (HPA) axis increases circulating glucocorticoids that have complex effects on neuroinflammation, synaptic plasticity and neuronal survival (Taheri et al., 2022). At the same time, the intracellular stress responses—including the unfolded protein response (UPR) and oxidative stress signaling—are juxtaposed with apoptotic and autophagic pathways. In case of their dysregulation, these responses intensify the secondary injury and limit the availability of neural repair (Yang et al., 2024). New data also indicate that specific stress-related mechanisms could further be manipulated to affect plasticity and promote cognitive improvement, which is why innovative, personalized treatments are justified (Bano et al., 2025).

### 1.1 Types of cellular stress in craniocerebral injury

**Figure 1:**
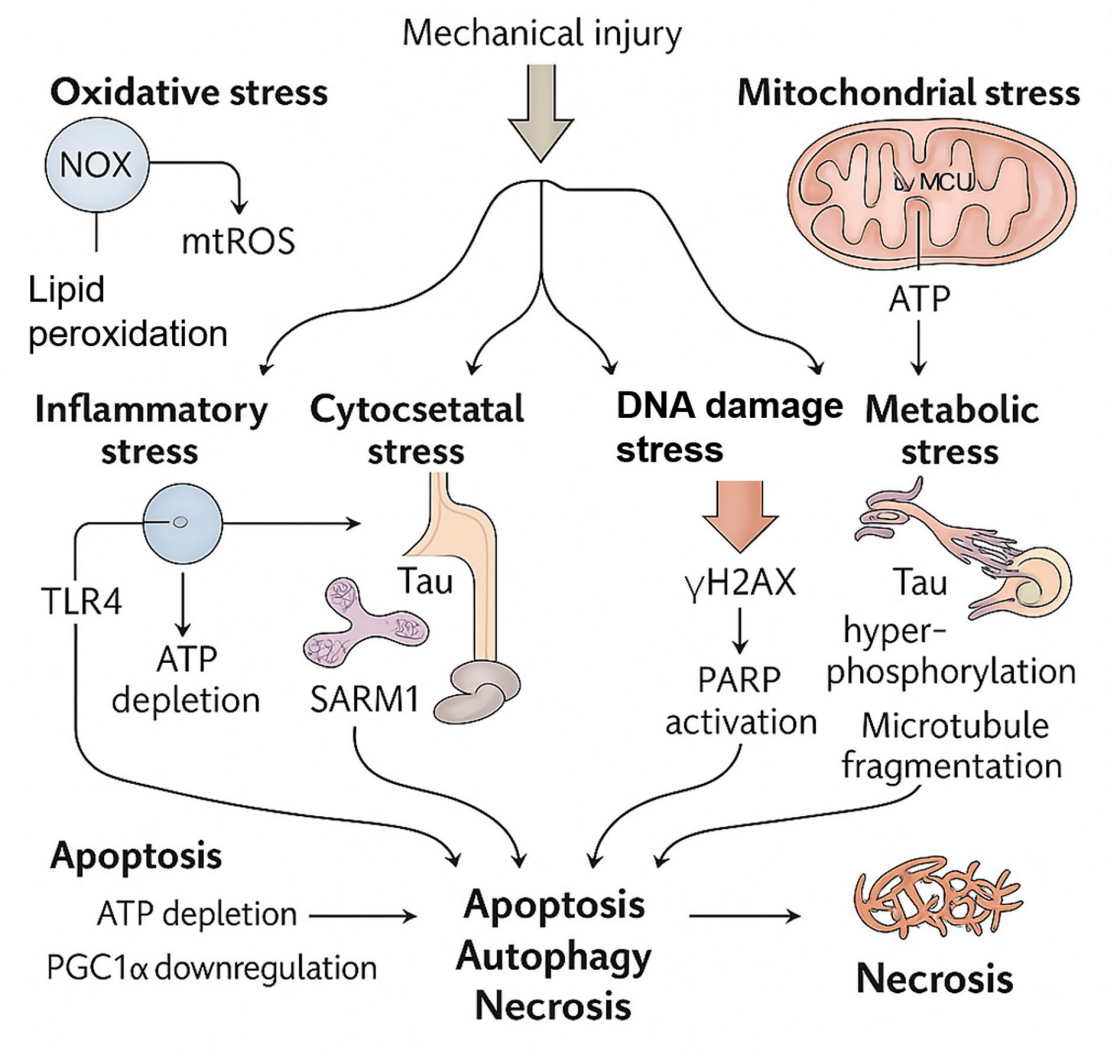
Types of cellular stress in craniocerebral injury.

Following TBI, neurons are subjected to multiple stressors, including mechanical stress, oxidative stress, and ER stress, triggering a cascade of pathological events that ultimately lead to neurological dysfunction (Yang et al., 2024). Axonal damage and cytoskeletal disruption activate Calpain-2, promoting neuronal apoptosis (Baudry et al., 2023). Meanwhile, oxidative and ER stress further compromise mitochondrial homeostasis, leading to calcium overload, mitochondrial DNA (mtDNA) damage, and activation of inflammasomes such as NOD-like receptor family pyrin domain-containing 3 (NLRP3) (Chakraborty et al., 2023). These stress signals stimulate the NF-κB pathway and promote the release of pro-inflammatory cytokines including interleukin-1β (IL-1β), interleukin-6 (IL-6), and tumor necrosis factor-*α* (TNF-α), thereby driving neuroinflammation and neuronal death (Caceres et al., 2024) (Figure 1).

NOX (NADPH oxidase); mtROS (mitochondrial reactive oxygen species); Ca^2+^ (calcium ion); MCU (mitochondrial calcium uniporter); mPTP (mitochondrial permeability transition pore); TLR4 (Toll-like receptor 4); NLRP3 (NACHT, LRR and PYD domains-containing protein 3); ATP (adenosine triphosphate); Tau (microtubule-associated protein Tau); SARM1 (sterile alpha and TIR motif-containing protein 1); γH2AX (phosphorylated H2A histone family member X); PARP (poly-ADP ribose polymerase); ATM (ataxia-telangiectasia mutated); ATR (ataxia-telangiectasia and Rad3-related protein); PGC1α (peroxisome proliferator-activated receptor gamma coactivator 1-alpha).

TBI precipitates an abrupt and severe escalation in ROS and reactive nitrogen species (RNS), including superoxide (O₂•^−^), hydrogen peroxide (H₂O₂), hydroxyl radical (•OH), nitric oxide (NO), and peroxynitrite (ONOO^−^) (Fesharaki-Zadeh, 2022). These radicals emerge from multiple interacting sources—mitochondrial electron transport chain leakage, activation of Nicotinamide Adenine Dinucleotide Phosphate (NADPH) oxidase (NOX), particularly NOX2 in microglia and neurons via subunits like p47^phox, p67^phox and Rac2—and excitotoxic cascades driven by glutamate overstimulation. The result is an early and overwhelming oxidative/nitrosative load that disrupts the Reduction–Oxidation Reaction (REDOX) equilibrium in neural tissue. As mitochondrial function deteriorates due to Ca^2+^ overload and membrane potential collapse, there is both reduced ATP synthesis and increased ROS release, establishing a vicious feedback cycle that amplifies oxidative damage and cellular energetic failure (Modi et al., 2024). Importantly, recent evidence shows that oxidative stress is closely linked to ER–mitochondria contact dynamics (MAMs), as Ca^2+^ miscommunication and mitochondrial depolarization directly intensify ROS production after TBI, reinforcing the central role of MAM remodeling during early secondary injury.

The downstream effects of excess ROS/RNS include lipid peroxidation—generating cytotoxic aldehydes like 4-hydroxynonenal (4-HNE), malondialdehyde (MDA), and isoprostanes—oxidative modifications of proteins, and DNA strand breaks (including oxidized bases and double-strand breaks). This molecular injury compromises membrane integrity, disrupts the blood–brain barrier (BBB), causes neuronal and endothelial apoptosis or necrosis, and potentiates neuroinflammation and edema formation (Kumari et al., 2025). Activation of nitric oxide synthases (especially iNOS), driven by calcium influx through NMDA/AMPA receptors in glutamate excitotoxicity, significantly raises NO levels which rapidly combine with superoxide to form peroxynitrite—intensifying nitrosative stress and further damaging mitochondria, DNA and cytoskeleton (Ryan et al., 2023; Prolo et al., 2024). The endogenous antioxidant defense system—comprising superoxide dismutase (SOD), catalase, glutathione peroxidase (GPx), and glutathione synthesis—is overwhelmed or downregulated post-injury, rendering cells unable to counteract the surge of ROS/RNS effectively (Baudry et al., 2023). Moreover, ROS and pro-inflammatory cytokines such as IL-1β, IL-6 and TNF-*α* engage in a mutually reinforcing loop: ROS activates NF-κB and the NLRP3 inflammasome, which elevate cytokine release; in turn, inflammation further induces NOX expression and ROS production, perpetuating secondary injury cascades (Wang et al., 2023).

In concert, these overlapping mechanisms—mitochondrial dysfunction, glutamate excitotoxicity, NOX-mediated ROS generation, antioxidant collapse, lipid/protein/DNA oxidation, and inflammatory signaling—constitute the central oxidative/nitrosative stress axis in TBI (Fesharaki-Zadeh, 2022). The uniquely lipid-rich and high-metabolism characteristics of brain tissue make it exceptionally susceptible to oxidative damage, escalating sensorimotor dysfunction, synaptic disruption, diffuse axonal injury, and long-term neurodegeneration (Ryan et al., 2023). Notably, oxidative stress also modulates MAM-associated Ca^2+^ transfer and ER stress sensors, positioning redox imbalance as an upstream driver of later mitochondrial–ER stress crosstalk described in Section 2.

In the last 5 years, both preclinical and early clinical investigations have spotlighted several antioxidant strategies aiming to intercept the oxidative/nitrosative cascade following TBI (Tabet et al., 2022). N-acetylcysteine (NAC), a glutathione precursor and thiol antioxidant, has been tested in early clinical contexts: in a randomized controlled pilot study in moderate to severe TBI patients, high-dose NAC administered within hours of injury significantly reduced serum markers such as MDA, IL-6, S100B and NSE, shortened ICU stay, and improved Glasgow Coma Scale scores (*p* < 0.001) relative to placebo (Gouda et al., 2025). These findings suggest that NAC offers multimodal benefits—ROS scavenging, anti-inflammatory, and neuroprotective actions, possibly via NF-κB inhibition and restoration of REDOX and glutamatergic homeostasis (Clark et al., 2023). However, its effectiveness remains time-sensitive, and larger multicenter trials are still needed to confirm benefit across diverse TBI severities.

Synthetic superoxide dismutase mimetics (SOD mimetics), including manganese-based catalytic compounds such as M40403/M40401 and Mn-salen/Mn-porphyrin complexes, have advanced in cellular and animal models; they catalyze superoxide dismutation with longer half-lives and better cell/BBB permeability than the native enzyme. *In vitro* and *in vivo* studies show reductions in lipid peroxidation, suppression of inflammatory gene expression, and improved neuronal survival under oxidative challenge (Grujicic and Allen, 2024). Yet, despite strong preclinical promise, the translation of SOD mimetics into TBI clinical studies remains limited, and their potential off-target effects—including interactions with physiological NO signaling—remain concerns for long-term safety.

Further, recent computational protein engineering has produced hyper-stable SOD variants from radiation-resistant *Deinococcus* species with enhanced catalytic efficiency and thermostability, highlighting next-generation biologics for oxidative modulation in TBI/ischemia–reperfusion contexts (Furukawa et al., 2025). These biologics remain in preclinical stages, and their relevance to human TBI still requires cautious interpretation, as most evidence derives from non-TBI oxidative injury models.

Another focus is mitochondrial-targeted antioxidants such as MitoQ and edaravone. MitoQ accumulates in mitochondria, activates antioxidant defenses (e.g., Nrf2/ARE), improves mitochondrial integrity, and enhances behavioral recovery in rodent TBI models (Tabet et al., 2022). Edaravone, a clinically used radical scavenger for stroke, also shows protective effects in preclinical TBI settings through REDOX-modulating and anti-inflammatory mechanisms (Li et al., 2023). However, differences in pharmacokinetics, BBB penetration, and optimal timing may contribute to variable therapeutic outcomes, highlighting the need to define patient-specific therapeutic windows. Reviews of mitochondrial-targeted therapies emphasize their translational potential but also highlight challenges such as delivery, dosing/timing, and patient heterogeneity (Modi et al., 2024).

More recently, nanotechnology-mediated combination therapies are gaining traction. For example, ROS-responsive nanoparticles (e.g., CAQK-modified PPS) loaded with curcumin have been developed to localize to injury sites, scavenge ROS, inhibit NF-κB–mediated inflammation, protect the BBB, reduce edema, and improve neurological outcomes in animal TBI models (Fu et al., 2025). Such multimodal agents attempt to break the ROS–inflammation vicious cycle via targeted, spatiotemporal intervention (Shi et al., 2024). Nonetheless, these findings remain largely preclinical, and nanoparticles introduce additional translational challenges including systemic clearance, potential immunogenicity, and manufacturing variability.

Current consensus shows the limitations of monotherapy: antioxidant cocktails combining NAC, SOD mimetics, mitochondrial agents, nanocarriers, and inflammatory pathway modulators are now advocated, aiming to act at multiple nodes of the oxidative/inflammatory cascade, improve BBB delivery, and extend the therapeutic window beyond the initial post-injury hours (Fesharaki-Zadeh, 2022). However, evidence supporting combination therapy is still mostly limited to animal models, and human validation is lacking. Given that TDP-43 mutations in ALS can alter stress-granule dynamics under oxidative stress and exacerbate ROS–inflammation cycles, such combinatorial approaches may also help mitigate TDP-43–associated cytoplasmic aggregation and downstream neurodegeneration (Ding et al., 2021). These cross-disease parallels should be interpreted cautiously, as direct evidence in TBI remains limited.

Nevertheless, clinical translation remains challenging: patient variability in injury severity, time to intervention, dosing regimens, and lack of standardized protocols contribute to inconsistent trial outcomes (Maas et al., 2022). No antioxidant therapy has yet achieved FDA approval for TBI (Fesharaki-Zadeh, 2022). Future progress hinges on well-powered, multi-center trials with stratified cohorts, refined biomarkers (e.g., oxidative stress panels, PNPT1/QDPR expression), and integrative therapeutic regimens to enable personalization of antioxidant interventions and improved functional recovery (Helmrich et al., 2022).

### 1.2 ER stress, ERS

TBI precipitates profound disturbances in intracellular calcium homeostasis and protein-folding capacity, leading to the activation of ER stress (ERS) and the UPR (Yang et al., 2024). Immediately following the primary mechanical insult, membrane disruption and excessive glutamate release provoke calcium influx into neurons and glial cells. Elevated cytosolic Ca^2+^ is sequestered by the ER, overwhelming its buffering capacity and inducing luminal calcium imbalance. This dysregulation impairs the ER’s protein-folding environment, resulting in the accumulation of misfolded and unfolded proteins—a hallmark trigger of the UPR (Wang et al., 2024). Notably, Ca^2+^ disequilibrium at ER–mitochondria contact sites (MAMs) further exacerbates ER stress and contributes to the propagation of secondary injury, linking ER dysfunction to mitochondrial depolarization and oxidative damage after TBI.

The UPR comprises three canonical signaling branches—Protein Kinase RNA-like Endoplasmic Reticulum Kinase (PERK)–Eukaryotic Initiation Factor 2 Alpha Subunit (eIF2α)–CHOP, Inositol-Requiring Enzyme 1 (IRE1)–X-box Binding Protein 1 (XBP1), and Activating Transcription Factor 6 (ATF6)—each orchestrating adaptive or apoptotic outcomes depending on stress severity and duration (Yang et al., 2024). In the PERK pathway, phosphorylation of eIF2α attenuates global protein translation to reduce ER load, while selectively inducing transcription factors such as ATF4 and CHOP. CHOP serves as a pro-apoptotic mediator, upregulating pro-death genes (e.g., BIM) and downregulating Bcl-2 survival proteins, ultimately promoting mitochondrial outer membrane permeabilization and caspase-3 activation (Hood et al., 2018). Concurrently, the IRE1 branch facilitates splicing of XBP1 mRNA, generating XBP1s, a transcription factor that augments ER chaperone expression and ER-associated degradation (ERAD). However, prolonged IRE1 activation can also recruit TRAF2 and ASK1, initiating JNK-mediated apoptosis and inflammatory amplification (Kang et al., 2024). The ATF6 arm, upon regulated intramembrane proteolysis, upregulates chaperones and folding enzymes; yet sustained ATF6 activation synergizes with CHOP to exacerbate neuronal death in severe TBI (Zhang et al., 2023). These findings underscore that UPR signaling displays strong temporal and context dependence—short-term activation may be adaptive, whereas prolonged activation drives apoptosis and neuroinflammation.

ERS and oxidative stress operate in a self-reinforcing loop: excess ROS generated from dysfunctional mitochondria and NOX enzymes impair protein folding capacity, while ER Ca^2+^ leakage—often occurring at MAMs—activates the NLRP3 inflammasome and accelerates IL-1β/IL-18 maturation (Chakraborty et al., 2023). Crosstalk between ER stress and autophagy is likewise bidirectional: early autophagy provides cytoprotection by clearing misfolded proteins, but chronic ER stress suppresses lysosomal function, contributing to cell death and glial scar formation (Wang et al., 2021). ERS markers such as GRP78/BiP, CHOP and p-eIF2α are elevated after TBI and correlate with injury severity in both rodent models and human biopsy/post-mortem studies. Collectively, ERS serves as a central molecular hub integrating Ca^2+^ dysregulation, proteostasis failure, oxidative injury, and neuroinflammation.

Given this pivotal role, pharmacological UPR modulation is being actively explored. Chemical chaperones such as 4-phenylbutyrate (4-PBA) and tauroursodeoxycholic acid (TUDCA) demonstrate neuroprotection in TBI models: 4-PBA enhances ER folding capacity and attenuates CHOP/caspase signaling with cognitive benefit in injury paradigms (Yang et al., 2024); TUDCA modulates PERK/IRE1 activity, limits apoptosis and pyroptosis, reduces edema/BBB disruption, and improves neurological recovery in controlled cortical impact models (Xu et al., 2025). However, these compounds exhibit relatively broad activity, and their long-term safety and optimal dosing windows in human TBI remain unclear.

Emerging small-molecule inhibitors and gene-directed approaches aim to selectively fine-tune UPR signaling. Partial PERK inhibition by compounds like GSK2606414 suppresses eIF2α phosphorylation and CHOP-driven apoptosis while attempting to preserve adaptive responses (Dhir et al., 2023). RNase-targeted inhibition of IRE1 via STF-083010 reduces XBP1 splicing and downstream JNK activation, dampening inflammatory signaling and glial reactivity in preclinical systems (Niu et al., 2021). Gene-directed modulation—such as CHOP suppression or GRP78 knockdown—illustrates the divergent functional roles of UPR components (Ha et al., 2024; Tian et al., 2021). Nonetheless, many of these findings originate from non-TBI neurological or oxidative-injury models, and their relevance to complex human TBI physiology should be interpreted cautiously. Off-target toxicity, metabolic burden, and UPR over-suppression remain key translational barriers.

Combining ER stress inhibitors with antioxidants (e.g., NAC, MitoQ) or anti-inflammatory drugs (e.g., minocycline) has shown greater efficacy than monotherapies (Davis et al., 2022). These multimodal strategies leverage the mechanistic interdependence among ER dysfunction, redox imbalance, mitochondrial impairment, and immune activation—core processes in TBI and multiple neurodegenerative diseases (Yang et al., 2024). Yet, combination therapy evidence remains predominantly preclinical, and optimal therapeutic timing has not been standardized.

Translational progress remains limited: no ERS-targeted agent has advanced beyond phase II clinical testing for TBI. Although TUDCA is FDA-approved for liver disease and is being assessed for broader neurological indications, rigorous TBI-specific trials are still needed (Khalaf et al., 2022). Biomarker development is advancing, with serum GRP78 and CHOP—together with neuroimaging (e.g., DTI) and electrophysiological indices—proposed for patient stratification and treatment monitoring. Future research emphasizes precision modulation of UPR signaling: transient enhancement of adaptive branches (IRE1-XBP1, ATF6) while restraining maladaptive PERK-CHOP signaling may optimize recovery without compromising physiological proteostasis. Integration of multi-omics and single-cell transcriptomics is expected to clarify cell-type-specific ER stress trajectories and guide personalized intervention strategies for TBI patients (Oris et al., 2024).

### 1.3 Mitochondrial stress

Mitochondria are central to neuronal energy metabolism and cell survival, and their dysfunction represents a pivotal driver of secondary injury in TBI (Modi et al., 2024). Both the initial mechanical insult and the ensuing secondary cascades—including excitotoxicity, calcium dysregulation, and oxidative stress—converge on mitochondrial failure (Qian et al., 2024). Within minutes to hours after trauma, mitochondrial membrane potential (ΔΨm) collapses due to excessive calcium influx and oxidative damage to respiratory chain complexes (particularly complexes I and III), leading to profound reductions in ATP synthesis (Manczak et al., 2011; Sun et al., 2022). Energy depletion impairs ion pumps, exacerbates cytotoxic edema, and promotes neuronal depolarization, thus amplifying glutamate release and perpetuating excitotoxic loops (Thapak and Gomez-Pinilla, 2024).

Mitochondrial calcium overload further triggers the opening of the mitochondrial permeability transition pore (mPTP), facilitating the release of pro-apoptotic factors such as cytochrome c and apoptosis-inducing factor (AIF) into the cytoplasm (Modi et al., 2024; Endlicher et al., 2023). Cytochrome c associates with Apaf-1 to form the apoptosome, activating caspase-9 and subsequently caspase-3, culminating in programmed neuronal death (Yang et al., 2024). Concurrently, mitochondrial production of ROS escalates under impaired electron transport, further damaging mitochondrial DNA (mtDNA), lipids, and proteins (Mira et al., 2023). Oxidative mtDNA lesions compromise respiratory chain function, establishing a vicious cycle of ROS amplification and energetic failure (Thapak and Gomez-Pinilla, 2024).

Beyond bioenergetic collapse, mitochondrial dynamics—the balance between fission and fusion—are profoundly disturbed in TBI (Tabassum et al., 2025). Proteins regulating fission (e.g., DRP1, Fis1) are upregulated, while fusion mediators (e.g., Mfn1/2, OPA1) are downregulated, resulting in fragmented mitochondrial networks (Anash et al., 2025). This morphological shift correlates with heightened apoptosis and synaptic dysfunction (Tabassum et al., 2025). Moreover, the interplay between mitochondrial dysfunction and autophagy/mitophagy is bidirectional: early mitophagy may eliminate damaged organelles and limit ROS, but persistent mitochondrial injury overwhelms autophagic flux, contributing to neuronal death and glial activation (Niu et al., 2019). Crosstalk with inflammatory signaling is evident—mtDNA released into the cytosol or extracellular space serves as a damage-associated molecular pattern (DAMP), activating pattern recognition receptors (e.g., TLR9, NLRP3) and amplifying neuroinflammation (Tian et al., 2025). Overall, mitochondrial stress integrates bioenergetic failure, oxidative damage, apoptotic signaling, and neuroimmune activation, making it a central mediator of secondary brain injury and a prime therapeutic target (O'Brien et al., 2020; Houle and Kokiko-Cochran, 2021).

Therapeutic strategies targeting mitochondrial dysfunction in TBI have advanced significantly in the last five years, encompassing approaches that enhance mitochondrial biogenesis, scavenge mitochondrial ROS, and modulate dynamics and mitophagy (O'Brien et al., 2020; Kalra et al., 2022). Augmenting mitochondrial biogenesis via the PGC1α (peroxisome proliferator-activated receptor gamma coactivator 1-alpha) pathway has shown particular promise (You et al., 2024). PGC1α acts as a master regulator of mitochondrial biogenesis by co-activating nuclear respiratory factors (NRF1/2) and mitochondrial transcription factor A (TFAM), thereby increasing mtDNA replication and respiratory enzyme expression (You et al., 2024). Pharmacological activators such as resveratrol, AICAR, and bezafibrate have demonstrated enhanced PGC1α expression, improved mitochondrial function, and reduced neurodegeneration in rodent TBI models (Placeres-Uray et al., 2025; Sun et al., 2020).

Mitochondria-targeted antioxidants represent another major therapeutic frontier (Modi et al., 2024). Molecules like MitoQ (mitoquinone) and 10-(6′-Plastoquinonyl) Decyltriphenylphosphonium (SkQ1), engineered to accumulate within mitochondria via lipophilic triphenylphosphonium cations, directly neutralize mitochondrial ROS and preserve membrane integrity (Fields et al., 2023). Preclinical studies reveal that MitoQ administration reduces oxidative damage markers (e.g., MDA, 4-HNE), inhibits cytochrome c release, and improves neurological outcomes in controlled cortical impact models (Haidar et al., 2022). Similarly, edaravone—although not mitochondria-specific—exerts substantial ROS-scavenging effects and has entered clinical use for stroke, suggesting potential repurposing for TBI (Wang et al., 2023).

Modulation of mitochondrial dynamics is emerging as an innovative therapeutic angle. Inhibitors of DRP1-mediated fission (e.g., Mdivi-1) have demonstrated reduced mitochondrial fragmentation, suppressed caspase activation, and improved cognitive recovery post-TBI (Wu et al., 2018).

Conversely, enhancing fusion via upregulation of major tethering proteins mitofusin-2 (MFN2) or OPA1 may restore network integrity and synaptic plasticity (Manczak et al., 2011). Targeting mitophagy is also under investigation: activation of PTEN-Induced Putative Kinase 1 (PINK1)/Parkin pathways facilitates selective removal of damaged mitochondria, while excessive mitophagy inhibition (e.g., using 3-MA) can worsen outcomes, highlighting the need for precise temporal modulation (Wu et al., 2018). Cutting-edge approaches combine mitochondrial interventions with other modalities to address the multifaceted nature of secondary injury. For instance, nanoparticles co-delivering MitoQ and anti-inflammatory agents have been engineered to cross the blood–brain barrier and synergistically mitigate oxidative stress and neuroinflammation (Sun et al., 2022).

Multi-target “cocktail” therapies integrating mitochondrial antioxidants, biogenesis enhancers, and ER stress modulators are now advocated to simultaneously tackle oxidative, inflammatory, and proteostatic dysfunctions (Davis et al., 2022). Despite compelling preclinical evidence, clinical translation remains limited: no mitochondrial-targeted therapy has yet achieved FDA approval for TBI, though MitoQ and related compounds are undergoing safety evaluations in other neurodegenerative contexts, providing a translational springboard for future trials (Khalaf et al., 2022). Ongoing research emphasizes precision medicine—using biomarkers such as circulating mtDNA, cytochrome c, or Peroxisome Proliferator-Activated Receptor Gamma Coactivator 1-Alpha (PGC1*α*) expression to stratify patients and optimize therapeutic timing and dosing (Sapin et al., 2021). These developments overall position mitochondrial stress modulation as a promising frontier in neuroprotective strategies for TBI (Modi et al., 2024; Sapin et al., 2021).

### 1.4 Inflammatory stress

TBI provokes a robust and multifaceted inflammatory response involving both the innate and adaptive immune systems (Obukohwo et al., 2024). Within minutes of the primary mechanical insult, resident immune cells—microglia and astrocytes—rapidly activate, undergoing morphological transformation and transcriptional reprogramming toward pro-inflammatory phenotypes (Shao et al., 2022). Activated microglia release an array of cytokines and chemokines, notably IL-1β, TNF-α, and IL-6, which orchestrate recruitment of peripheral immune cells, including neutrophils and monocytes, across a compromised BBB (Alam et al., 2020). This acute inflammatory milieu is initially protective, aiming to clear debris and promote repair, but becomes detrimental when excessive or prolonged, exacerbating neuronal loss and white matter damage (Shao et al., 2022). Importantly, early inflammatory activation is tightly linked to mitochondrial and ER dysfunction at ER–mitochondria contact sites (MAMs), where Ca^2+^ overload and ROS generation further potentiate inflammatory signaling.

At the molecular level, inflammatory stress is mediated by pattern recognition receptors (PRRs)—primarily Toll-like receptors (TLRs) and nucleotide-binding oligomerization domain-like receptors (NLRs)—which sense damage-associated molecular patterns (DAMPs) such as HMGB1, ATP, and extracellular mtDNA released from injured neurons (Li et al., 2023). TLR4 activation on microglia triggers MyD88-dependent signaling, culminating in NF-κB nuclear translocation and transcription of pro-inflammatory genes (e.g., TNF-α, IL-1β, IL-6) (Baral and Kaundal, 2025). Simultaneously, NLRP3 inflammasome assembly—comprising NLRP3, ASC, and pro-caspase-1—activates caspase-1, cleaving pro-IL-1β and pro–interleukin-18 (IL-18) into their mature, highly bioactive forms (O'Brien et al., 2020). This inflammasome-mediated cytokine surge amplifies BBB breakdown and recruits additional immune cells, perpetuating a vicious cycle of neuroinflammation (Li et al., 2024; Zhao et al., 2024; Qin et al., 2022). Notably, NLRP3 activation is strongly influenced by mitochondrial ROS, mtDNA leakage, and ER–mitochondria Ca^2+^ flux, linking inflammatory stress closely to upstream metabolic stressors characteristic of TBI.

Astrocytes complement microglial activity by releasing cytokines and chemokines (e.g., CCL2, CXCL1), modulating synaptic transmission and glial scar formation (Long et al., 2020). Moreover, crosstalk between inflammatory stress and oxidative/ER stress further aggravates neuronal injury: ROS produced by NADPH oxidase and damaged mitochondria potentiate NLRP3 activation, while ER stress–derived CHOP and calcium flux enhance NF-κB signaling (Liu et al., 2022). Chronic inflammation—characterized by persistent microglial activation and astrogliosis—drives secondary tissue degeneration, synaptic pruning, and progressive neurodegenerative changes akin to Alzheimer’s disease or chronic traumatic encephalopathy (Siracusa et al., 2025; Xu et al., 2023). Thus, inflammatory stress represents a double-edged sword in TBI, necessitating tightly regulated therapeutic modulation (Houle and Kokiko-Cochran, 2021).

The growing recognition of the detrimental role played by excessive neuroinflammation in TBI has led to the emergence of targeted anti-inflammatory therapies, with particular focus on inflammasome inhibition and microglial phenotypic modulation (Kalra et al., 2022). Among these, MCC950—a selective inhibitor of the NLRP3 inflammasome—has demonstrated neuroprotective effects in rodent models of mild traumatic brain injury (mTBI), especially under early-life stress (ELS). MCC950 suppressed up-regulation of NLRP3, caspase-1, and IL-1β mRNA in hippocampal microglia, improved glucocorticoid receptor signaling, and ameliorated fear memory deficits in the combined ELS + mTBI paradigm (Placeres-Uray et al., 2025). However, MCC950 has shown immune-suppression risks and hepatotoxicity in non-TBI models, highlighting translational challenges that require careful dose optimization and timing. In parallel, caspase-1 inhibitors such as VX-765 reduce inflammasome-dependent pyroptosis and cytokine release (Sun et al., 2020), though their short therapeutic window and systemic immunosuppression remain important considerations for clinical deployment.

Beyond direct inflammasome blockade, modulation of microglial phenotypes from pro-inflammatory M1 toward anti-inflammatory and reparative M2 states has gained traction (Li et al., 2023). Agents such as minocycline and CSF1R inhibitors shift microglial polarization, reduce neuroinflammation, and enhance axonal regeneration (Bergold et al., 2023). Despite promising results, both drug classes exhibit off-target effects, including interference with mitochondrial function or broad suppression of microglial populations, which may impair debris clearance in early TBI. Nanoparticle-based delivery systems co-encapsulating anti-inflammatory agents and antioxidants have been engineered to cross the BBB and provide spatiotemporal control of microglial activity (Fu et al., 2025). Still, nanoparticle stability, CNS biodegradation, and long-term safety require more rigorous evaluation before clinical translation.

Emerging therapies also target upstream inflammatory triggers: TLR4 antagonists (e.g., TAK-242) inhibit DAMP sensing; NF-κB inhibitors (e.g., parthenolide, BAY 11–7,082) blunt transcription of pro-inflammatory cytokines; and P2X7 receptor blockers suppress ATP-driven inflammasome activation (Feng et al., 2023). Combination therapies integrating these agents with antioxidants or ER stress modulators are under investigation to disrupt the mutually reinforcing cycle among inflammation, oxidative stress, and ER dysfunction (Li et al., 2023). However, many of these approaches derive supporting evidence from non-TBI inflammatory or neurodegenerative models, and direct relevance to human TBI physiology remains to be formally validated.

Despite promising preclinical results, clinical translation remains limited. No inflammasome-targeted drug has reached late-stage clinical trials for TBI, largely due to a narrow therapeutic window, patient heterogeneity, and risks of systemic immune suppression. Biomarker-driven approaches—using serum IL-1β, IL-18, or microglial PET imaging—may support patient stratification and guide precision immunomodulation (Visser et al., 2022). Future directions emphasize multi-modal interventions that target not only inflammatory mediators but also their mechanistic interplay with mitochondrial stress, oxidative injury, and ER dysfunction—an integrated strategy anticipated to yield more durable neuroprotection and functional recovery (Li et al., 2023).

### 1.5 Mechanical/cytoskeletal stress

TBI precipitates profound disruption of the neuronal cytoskeleton, a structural framework essential for maintaining axonal integrity, intracellular transport, and synaptic connectivity. Mechanical forces during the primary insult—including rapid acceleration-deceleration and rotational shear—directly cause axolemmal rupture, microtubule breakage, and neurofilament compaction, overall leading to diffuse axonal injury (DAI), a pathological hallmark of moderate-to-severe TBI (Grovola et al., 2021). DAI is characterized by axonal swellings, retraction bulbs, and impaired axonal transport, culminating in disconnection of neural networks and persistent cognitive deficits.

Central to cytoskeletal pathology is the calcium influx triggered by membrane disruption and glutamate excitotoxicity. Elevated intracellular Ca^2+^ activates calcium-dependent proteases, notably Calpain-2, which cleave critical cytoskeletal proteins such as microtubule-associated protein 2 (MAP2), spectrin, and neurofilament heavy chain (NF-H). This proteolysis destabilizes microtubules and intermediate filaments, further impairing axonal transport and precipitating synaptic degeneration (Schallerer et al., 2025). Concomitantly, microtubule depolymerization occurs due to post-traumatic oxidative modifications and hyperphosphorylation of tau proteins, which disrupt microtubule stability and axonal polarity (Jamjoom et al., 2021).

There is an emerging body of evidence that points to this multifactorial interaction exists between cytoskeleton stress and other pathways involved in cellular stress in TBI pathogenesis. The ROS derangement increases as a result of mitochondrial malfunction and as such oxidizes the tubulin and neurofilament subunits, enhancing cytoskeleton destruction. ER stress and neuroinflammation simultaneously exacerbate this damage by secreting pro-inflammatory cytokines, including TNF-*α* and interleukin-1 (IL-1). TNF-α and IL-1 activate the calcium-dependent protease Calpain-2, which degrades cytoskeleton proteins. Moreover, poor axonal transport causes the impaired supply of mitochondria and autophagosomes, which additionally affect cellular homeostasis and lead to the Wallerian degeneration (Yang et al., 2024). Together, these results indicate the issue of cytoskeletal stress as one of the primary interaction sites between mechanical harm and downstream biochemical signal cascades in TBI.

Drug treatments seeking to relieve damage to the cytoskeleton have targeted microtubule stabilization, Calpain-2 inhibition, and changing the control of cytoskeletal proteins post-translationally. Microtubule stabilizing drugs, like epothilone D (EpoD) and paclitaxel analogs have demonstrated potential to maintain axonal integrity and/or enhance axonal regeneration. In preclinical TBI models, EpoD treatment preserved the microtubule-associated protein 2 (MAP2), decreased axonal varicosities and substantially enhanced motor coordination and cognitive performance (Chuckowree et al., 2018). Concurrently, pharmacological inhibition of Calpain-2 enzyme with important medicines like calpeptin and SNJ-1945 followed a similar pattern of diminishing the proteolytic defragmentation of spectrin and MAP2, which helped in lessening axonal destruction and intensifying neurological healing (Bains et al., 2013). Notably, Calpain-2 and caspase pathways may be synergistically blocked to confer neuroprotective effects simultaneously and indicate the convergence of apoptotic and necrotic events in cytoskeletal breakdown. Other methods involve inhibiting tau phosphorylation using glycogen synthase kinase-3 (GSK-3) inhibitors, such as lithium and tideglusib, to prevent cross-linking of microtubules and tau aggregation. Moreover, mitochondrial-targeted antioxidants (e.g., MitoQ) as well as activators of the Nrf2 signaling pathway have been found as supplementary treatments to dampen oxidative stress-mediated cytoskeleton oxidation, especially when given together with the anti-excitiotoxics (Yang et al., 2024).

Nanotechnology-made drug delivery mechanisms have been used to increase drug distribution through the BBB and also to release drugs to the injury site within the axons (Mohammed et al., 2023). As an illustration, nanoparticle-coated Calpain-2 inhibitors or microtubule stabilizers generate local-prolonged concentrations of the drug, decreasing the overall intoxication and maximizing therapeutic effect (Madias et al., 2024). Despite these advances, clinical translation remains nascent: variability in injury biomechanics, timing of intervention, and off-target effects pose significant hurdles (Mohammed et al., 2023). Future research is oriented toward precision therapies—integrating biomarkers (e.g., serum neurofilament light chain, MAP2 fragments) and advanced neuroimaging (DTI, PET) to stratify patients and personalize cytoskeletal-targeted interventions (Shahim et al., 2020).

### 1.6 DNA damage stress

TBI triggers profound genomic instability, primarily through ROS overproduction and calcium dysregulation arising from excitotoxicity and mitochondrial failure (Zhang et al., 2024). Excess ROS—including hydroxyl radicals, superoxide, and peroxynitrite—induces oxidative base lesions (e.g., 8-oxo-guanine), single- and double-strand DNA breaks (DSBs), and DNA–protein crosslinks (Hahm et al., 2022). Simultaneously, intracellular calcium overload activates endonucleases and apoptotic nucleases, compounding DNA fragmentation and chromatin condensation (Neuschmid et al., 2025). Post-mortem and animal studies consistently report elevated markers of DNA damage, such as phosphorylated H2AX (γH2AX) foci and 8-oxoG accumulation, within hours to days after injury (Al-Khateeb et al., 2024).

The cellular response to DNA lesions is orchestrated by the DNA damage response (DDR) network, predominantly mediated by ATM and ATR kinases (Delint-Ramirez and Madabhushi, 2025). ATM is primarily activated by DSBs, recruiting downstream effectors such as Chk2 and p53 to regulate cell-cycle arrest and promote homologous recombination repair (Zhao et al., 2024). ATR responds to replication stress and single-stranded DNA, activating Chk1 and facilitating nucleotide-excision or base-excision repair pathways (Delint-Ramirez and Madabhushi, 2025). If damage is irreparable, ATM/ATR signaling shifts toward apoptosis, largely via p53-mediated transcription of pro-apoptotic genes (Bax, Puma) and mitochondrial outer-membrane permeabilization (Zhao et al., 2024). In neurons and glia, this delicate balance between repair and death dictates survival outcomes and determines the extent of secondary neurodegeneration (Delint-Ramirez and Madabhushi, 2025).

Notably, DNA damage also intersects with other stress pathways in TBI. Oxidative stress exacerbates DNA oxidation, while ER stress–induced CHOP activation and inflammatory mediators (e.g., TNF-*α*) potentiate p53-driven apoptosis (Qin et al., 2022). Mitochondrial dysfunction further propagates ROS, fueling a self-amplifying cycle of DNA injury. Because Ca^2+^ overload and ROS production often originate at ER–mitochondria contact sites (MAMs), DNA damage is increasingly viewed as a downstream integrator of MAM-dysregulated stress responses in TBI. Importantly, chronic insufficiency in DNA repair mechanisms contributes to long-term cognitive deficits and predisposes to neurodegenerative disorders such as Alzheimer’s disease and chronic traumatic encephalopathy, in which persistent γH2AX and PARP activation have been observed (Siracusa et al., 2025). These cross-disease findings highlight shared mechanisms but require cautious interpretation, as not all processes have been validated directly in TBI.

Therapeutic strategies aimed at mitigating DNA damage in TBI focus on enhancing DNA repair, scavenging ROS, and modulating DDR signaling (Davis and Vemuganti, 2021). Pharmacological activation of DNA repair pathways has gained traction: agents boosting base-excision repair (BER) or non-homologous end joining (NHEJ)—such as nicotinamide and PARP modulators—improve neuronal survival and functional outcomes in preclinical models (Salech et al., 2020). Importantly, PARP inhibitors (e.g., olaparib) show dual benefits: reducing energy depletion from PARP overactivation and promoting controlled repair, thereby preventing parthanatos-like cell death (Sun et al., 2022). Nevertheless, most PARP-related evidence derives from oncology and ischemia models; off-target metabolic effects and concerns regarding long-term genomic stability must be addressed before application in TBI.

ATM/ATR modulators present another promising avenue. Low-dose ATM activators enhance DSB repair capacity, whereas ATM/ATR inhibitors may reduce excessive p53-mediated apoptosis during acute phases. However, therapeutic timing is critical—early inhibition may preserve neurons, while prolonged suppression risks accumulation of unrepaired lesions and increased genomic instability (Xu et al., 2023). Experimental studies highlight context-dependent outcomes, emphasizing the need for precise temporal control.

Antioxidant strategies indirectly attenuate DNA damage by quenching ROS upstream (Modi et al., 2024). Compounds such as MitoQ and NAC reduce oxidative DNA lesions and synergize with DDR enhancers (Sun et al., 2022). Combination therapies incorporating antioxidants with DDR-targeting agents or neuroinflammation inhibitors (e.g., MCC950) show additive neuroprotection, underscoring the interconnected nature of stress responses in TBI (O'Brien et al., 2020). Still, translation is limited by variable BBB penetration, narrow therapeutic windows, and sparse human data.

Emerging approaches explore gene editing and epigenetic modulation (Salomonsson and Clelland, 2024). CRISPR-based repair templates targeting ATM/ATR mutations or base editing to correct oxidative lesions are in early-stage research, while epigenetic regulators (e.g., HDAC inhibitors) enhance chromatin accessibility for repair complexes (Palomes-Borrajo et al., 2025). Biomarker-driven strategies are also advancing: circulating γH2AX, 8-oxoG, and phosphorylated ATM/ATR serve as prognostic indicators and therapeutic response markers in clinical and preclinical studies (Davis and Vemuganti, 2021).

Despite these advances, clinical translation faces challenges: heterogeneity of TBI pathology, narrow therapeutic windows, and potential oncogenic risks of DDR modulation necessitate cautious optimization (Salomonsson and Clelland, 2024). Future directions focus on precision therapeutics—integrating single-cell transcriptomics and longitudinal biomarker profiling to tailor DNA repair–enhancing or apoptosis-suppressing strategies to individual patients (Shi et al., 2024). Ultimately, targeting DNA damage stress alongside oxidative, mitochondrial, and inflammatory pathways may provide a synergistic framework for comprehensive neuroprotection in TBI (Modi et al., 2024)

### 1.7 Metabolic stress

TBI induces profound metabolic derangements in the brain, characterized by a mismatch between energy demand and supply during both acute and chronic phases of injury (Fernandez-Gajardo et al., 2014). The mechanical insult triggers a hypermetabolic state, wherein ionic pump activity (notably Na^+^/K^+^ ATPase) and excitatory neurotransmitter release sharply elevate energy requirements (Thapak and Gomez-Pinilla, 2024). However, concomitant mitochondrial dysfunction and cerebral blood flow reduction restrict oxidative phosphorylation, precipitating a shift toward anaerobic glycolysis (Weng et al., 2025). This metabolic shift results in lactate accumulation, acidosis, and energy depletion (ATP decline), exacerbating neuronal excitability and promoting secondary injury cascades (Zeng et al., 2020).

A key feature of metabolic stress is increased glucose dependence and impaired utilization of alternative energy substrates. Under physiological conditions, neurons and astrocytes flexibly switch between glucose, lactate, and ketone bodies; post-TBI, however, glucose uptake rises while metabolic flexibility diminishes, partly due to altered expression of glucose transporters (GLUT1/3) and lactate shuttling mechanisms (MCT1/2). Proton accumulation from lactate contributes to intracellular acidosis, which disrupts ion channel function, exacerbates Ca^2+^ influx, and sensitizes neurons to excitotoxic damage (Zeng et al., 2020).

Metabolic stress is tightly interlinked with mitochondrial dysfunction and calcium homeostasis. Impaired oxidative phosphorylation in damaged mitochondria diminishes ATP generation and promotes ROS production; simultaneously, calcium overload further inhibits key mitochondrial dehydrogenases (e.g., pyruvate dehydrogenase, *α* ketoglutarate dehydrogenase), reinforcing the energy deficit (Thapak and Gomez-Pinilla, 2024). This vicious cycle contributes to axonal transport failure, synaptic loss, and glial activation. Chronic metabolic insufficiency is implicated in long-term cognitive impairment and neurodegenerative sequelae post-TBI, including Alzheimer’s-like pathology (Fernandez-Gajardo et al., 2014).

Therapeutic strategies addressing metabolic stress in TBI focus on restoring energy homeostasis, modulating substrate utilization, and protecting mitochondrial metabolism (Filippone et al., 2022). One major avenue is lactate supplementation: exogenous lactate serves as an alternative energy substrate, bypassing impaired glycolysis and supporting neuronal recovery (Guo et al., 2024). Preclinical studies demonstrate that lactate administration reduces lesion volume, mitigates acidosis, and improves behavioral outcomes by fueling oxidative metabolism in surviving mitochondria (Chen et al., 2024; Yang et al., 2024).

Another promising strategy involves ketone body supplementation or ketogenic diets. Ketones (*β* hydroxybutyrate, acetoacetate) provide efficient ATP production and exhibit intrinsic anti-inflammatory and antioxidant properties. Rodent TBI models show that ketogenic therapy enhances mitochondrial biogenesis, reduces ROS generation, and improves neurological function (Feng et al., 2022). Clinical pilot trials also suggest feasibility and safety of ketogenic nutrition in acute brain injury, though larger randomized studies are warranted (Zhao et al., 2023).

Mitochondria-targeted interventions play a dual role in alleviating metabolic stress: agents such as MitoQ and coenzyme Q10 improve oxidative phosphorylation efficiency and reduce ROS-mediated metabolic inhibition (Filippone et al., 2022). Activation of PGC 1α signaling boosts mitochondrial biogenesis and restores energy balance, while pharmacological enhancers (e.g., bezafibrate, resveratrol) demonstrate neuroprotection in TBI models (Modi et al., 2024). Calcium modulation therapies indirectly ameliorate metabolic stress by preventing calcium-induced mitochondrial enzymatic inhibition. Calcium channel blockers (nimodipine), mPTP inhibitors (cyclosporin A), and SERCA activators have been explored for preserving mitochondrial bioenergetics. Also, metabolic monitoring tools (microdialysis, ^31P MRS) are being integrated in clinical neurocritical care to tailor interventions based on real-time lactate/pyruvate ratios and cerebral metabolic profiles (Filippone et al., 2022).

Despite promising advances, translation to clinical practice remains challenging: inter-individual metabolic variability, timing of intervention, and nutritional considerations require precision approaches. Future strategies will likely combine multi-substrate therapies (lactate + ketones), mitochondria-targeted antioxidants, and real-time metabolic biomarkers to personalize treatment and optimize outcomes.

## 2 The molecular mechanism of mitochondrial-ER stress crosstalk

**Figure 2:**
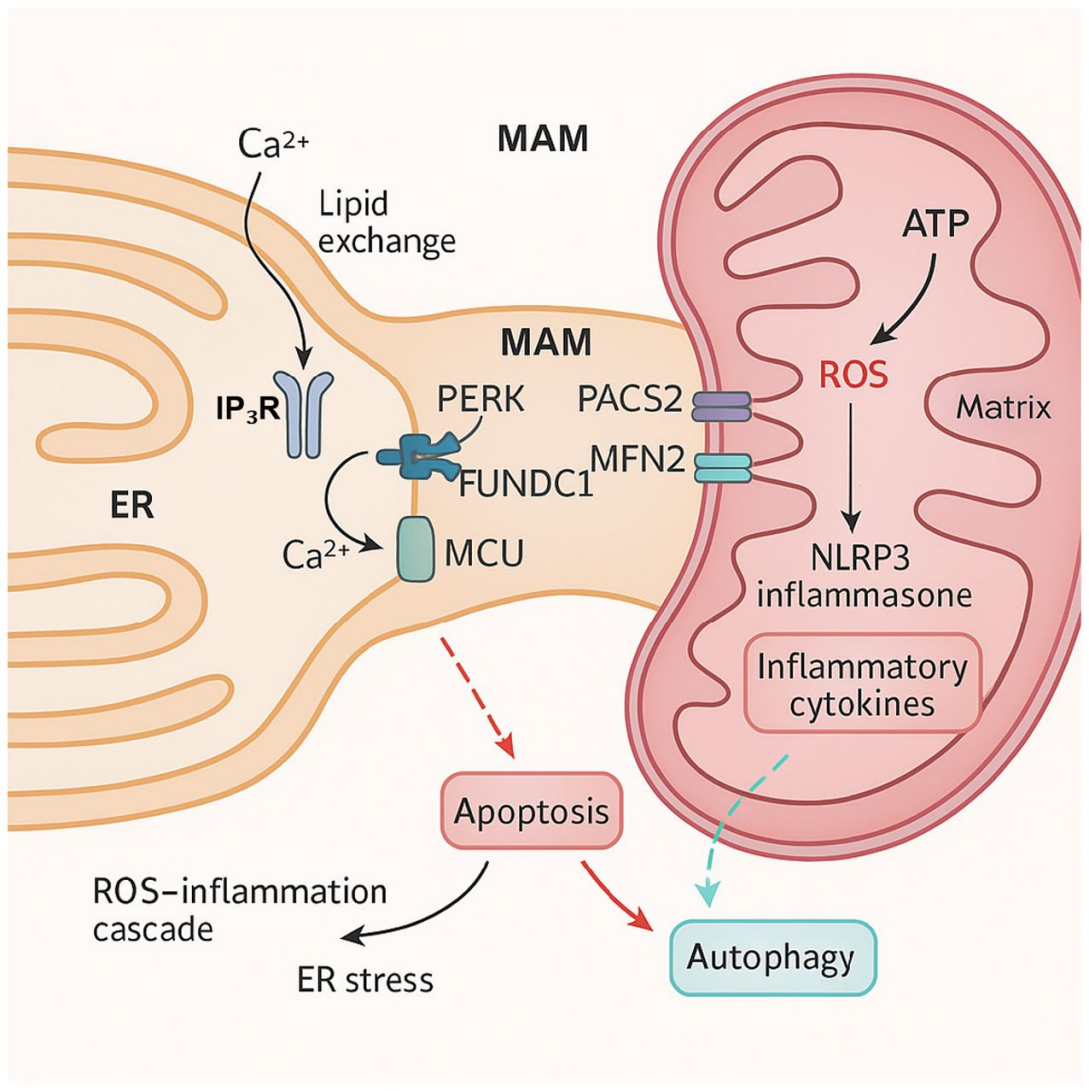
The molecular mechanism of mitochondrial-ER stress crosstalk. ER, endoplasmic reticulum; MAM, mitochondria-associated membrane; IP₃R, inositol 1,4,5-trisphosphate receptor; Ca^2+^, calcium ion; MCU, mitochondrial calcium uniporter; PERK, protein kinase RNA-like endoplasmic reticulum kinase; PACS2, phosphofurin acidic cluster sorting protein 2; MFN2, mitofusin 2; FUNDC1, FUN14 domain-containing protein 1; ATP, adenosine triphosphate; ROS, reactive oxygen species; NLRP3, NACHT, LRR and PYD domains-containing protein 3; inflammasome.

In cellular stress responses and metabolic disorder-related diseases—such as neurodegenerative diseases, cancer, and metabolic syndrome—the functional crosstalk between the ER and mitochondria has become a major research focus. Under stress conditions, ER stress significantly affects mitochondrial function through calcium (Ca^2+^) signaling, ROS production, and structural remodeling of MAMs, ultimately leading to autophagy, apoptosis, and inflammatory responses (Figure 2).

### 2.1 MAMs structure and function

Mitochondria-associated ER membranes (MAMs) are specialized subdomains of the ER that maintain tight and highly regulated contacts with the outer mitochondrial membrane, enabling direct inter-organelle communication. Recent work indicates a large and multifunctional proteome—on the order of 10^3^ proteins—and dynamic coverage of the mitochondrial surface by ER contacts (typically 5–20%), supporting substantial structural heterogeneity (Zhao and Sheng, 2025; Pihan et al., 2025). Proteomic and ultrastructural studies further underscore this complexity, identifying ~1,300 MAM-enriched proteins and revealing age-, injury-, and context-dependent remodeling of contact coverage (Lu et al., 2022).

Functionally, lipid-metabolic enzymes enriched at MAMs (e.g., ACAT1/SOAT1, phosphatidylserine synthases, DGATs) highlight their central role as lipid-biosynthetic and lipid-exchange hubs. Perturbing ACAT1/SOAT1 alters local cholesterol composition and strengthens ER–mitochondria connectivity (Harned et al., 2023; Long et al., 2024). These observations originate largely from metabolic and neurodegenerative models, but similar lipid-dependent regulation of MAM architecture is emerging in TBI, where membrane disruption and phospholipid turnover are markedly altered.

Calcium (Ca^2+^) homeostasis at Mitochondria–Endoplasmic Reticulum Contact Sites (MERCS) is regulated by structural tethers such as MFN2, which couples with SERCA2 to fine-tune Ca^2+^ transfer and mitochondrial metabolism (Yang et al., 2023). In TBI, MFN2 expression and post-traumatic Ca^2+^ flux are dysregulated, leading to excessive mitochondrial Ca^2+^ loading that sensitizes the permeability transition pore and enhances ROS production—two key drivers of secondary injury. Thus, MAM-resident Ca^2+^-handling complexes serve as critical amplifiers of the abnormal Ca^2+^ signals triggered by mechanical injury, glutamate excitotoxicity, and membrane rupture.

Finally, the ER stress sensor PERK localizes to ER–mitochondria contacts and participates in stress transduction that links ER proteostasis to mitochondrial dysfunction and apoptosis-related pathways (Sassano et al., 2023). Given that ER stress, oxidative stress, and Ca^2+^ dysregulation co-occur early after TBI, PERK positioning at MAMs helps explain how ER-derived signals rapidly propagate to mitochondria to induce depolarization, bioenergetic collapse, and CHOP-dependent apoptosis.

Together, these insights position MAMs as dynamic integrators of metabolic, proteostatic, and stress-response signaling within ER–mitochondrial communication. Importantly, although much of the mechanistic detail originates from non-TBI models (e.g., metabolic disease, AD, ischemia), accumulating evidence suggests that post-traumatic alterations in MAM architecture and Ca^2+^ handling play a key role in coordinating the early secondary injury cascades characteristic of TBI.

### 2.2 Ca^2+^ transport and energy metabolism dysregulation

MAMs are specialized domains where the ER and mitochondria are physically linked to form discrete sites of organelle contact (Larranaga-SanMiguel et al., 2025). This localization has important functional implications because the narrow intermembrane gaps (10–30 nm wide) are required to enable efficient calcium (Ca^2+^) transfer and are central to the control of both mitochondrial energy metabolism and apoptotic signaling cascades (Zhang et al., 2024). In TBI, membrane rupture, glutamate excitotoxicity, and intracellular Ca^2+^ surges disproportionately burden these MAM contact sites, making them critical early amplifiers of post-traumatic metabolic dysfunction.

The molecular regulation of calcium flow across MAMs involves a core triad: inositol 1,4,5-trisphosphate receptor (IP₃R) on the ER membrane, the molecular chaperone glucose-regulated protein 75 (GRP75), and the voltage-dependent anion channel 1 (VDAC1) on the outer mitochondrial membrane. This IP_3_R–GRP75–VDAC1 bridge establishes a direct Ca^2+^ conduit between the ER and mitochondria, facilitating rapid and targeted calcium signaling (Meng et al., 2023). Dysregulation of this complex has been observed in several neurological injuries, and accumulating evidence suggests similar alterations after TBI, where excessive IP_3_R-mediated Ca^2+^ release contributes to mitochondrial overload.

Upon reaching the mitochondrial surface, Ca^2+^ is transported into the matrix via the mitochondrial calcium uniporter (MCU), a highly regulated multi-protein complex comprising the pore-forming MCU subunit and regulatory partners MICU1, MICU2, and EMRE (Essential MCU Regulator) (Huo and Molkentin, 2024). The activity of this uniporter is tightly controlled, depending on localized high Ca^2+^ concentrations at the ER–mitochondria interface and the maintenance of mitochondrial membrane potential (Nava Lauson et al., 2023). In TBI, both of these regulatory conditions—Ca^2+^ microdomains and membrane potential—are disrupted, which lowers the threshold for pathological mitochondrial Ca^2+^ uptake.

Moderate calcium influx activates tricarboxylic acid (TCA) cycle enzymes such as pyruvate dehydrogenase, isocitrate dehydrogenase, and *α*-ketoglutarate dehydrogenase, thereby enhancing production of NADH and FADH₂ to fuel oxidative phosphorylation and ATP synthesis (Trushina et al., 2022). Under normal physiological conditions, this coordinated Ca^2+^ transfer boosts metabolic efficiency and supports cellular energy demands (Fu et al., 2025).

However, excessive or prolonged Ca^2+^ influx can lead to mitochondrial calcium overload, triggering opening of the mitochondrial permeability transition pore (mPTP) (Narmashiri et al., 2022), loss of the inner membrane potential, ATP depletion, ROS overgeneration, and activation of intrinsic cell death pathways (Wang et al., 2024). In the context of TBI—where Ca^2+^ surges coincide with membrane depolarization, oxidative stress, and ER dysfunction—mPTP opening occurs earlier and more robustly, driving rapid metabolic collapse and neuronal vulnerability.

Consequently, while MAM-mediated Ca^2+^ signaling is essential for cellular energetics and survival, its dysregulation transforms it into a pathogenic driver of mitochondrial dysfunction, ER stress propagation, and neurodegeneration. Emerging studies increasingly support the view that post-traumatic remodeling of the IP₃R–GRP75–VDAC1–MCU axis constitutes a central mechanism linking mechanical injury to the metabolic crisis characteristic of TBI.

### 2.3 ROS and inflammatory cascade reactions

ROS generated by mitochondria (mtROS) serve as both intracellular messengers and pro-inflammatory triggers (Yan et al., 2022). Leakage of electrons from respiratory complexes I and III during oxidative phosphorylation forms superoxide, rapidly converted by mitochondrial dismutases SOD2 and SOD1 into hydrogen peroxide. Under physiological conditions, low-level mtROS facilitate REDOX signaling—for instance, triggering hypoxia adaptation or priming innate immune responses via TLRs (Wang et al., 2020).

However, when mitochondrial function is compromised—whether due to electron transport chain (ETC) dysfunction, loss of MAM structural integrity, or persistent ER stress—the balance shifts toward excessive accumulation of mtROS (Mohan and Talwar, 2025). In TBI, Ca^2+^ overload, mechanical membrane disruption, and abrupt metabolic failure create an environment in which MAM-dependent Ca^2+^ microdomains and impaired ETC activity rapidly elevate mtROS beyond physiological buffering capacity. This early surge overwhelms endogenous antioxidant defenses and accelerates secondary injury.

A case in point is the downregulation of a major tethering protein MFN2 localized to MAMs (Yang et al., 2023). Loss of MFN2 impairs ER–mitochondrial coupling, resulting in increased ROS production, exacerbated ER stress, and induction of apoptosis in epithelial cells (Gottschalk et al., 2022). Although these findings originate largely from non-TBI systems, similar patterns of MFN2 loss and MAM uncoupling have been observed after traumatic injury, suggesting a conserved mechanism contributing to mtROS escalation in TBI.

Moreover, accrued mtROS can act as danger-associated molecular patterns (DAMPs), stimulating innate immunity receptors and amplifying downstream inflammatory signaling in a self-amplifying feedback loop (Ma et al., 2024). Cytokine-induced hyperproduction of mtROS acts as a potent NLRP3 inflammasome activator, a decisive component of innate immunity (Yu et al., 2024). This mechanism is highly relevant to TBI, where NLRP3 activation is consistently reported in both acute and chronic phases, correlating with neurological deficits and glial activation.

In the context of mitochondrial distress, MAMs constitute specialized platforms where key inflammasome components—together with adapters such as MAVS—assemble to stimulate IL-1β production and release of pro-inflammatory cytokines. Thus, mtROS generation and the spatial organization of inflammasome machinery converge at MAMs, making these contact sites a structural amplifier of neuroinflammation following TBI.

ROS also engage REDOX-sensitive signaling routes—such as MAPKs and ERK1/2—that transduce stress signals into cytokine production and inflammatory gene expression (Martin-Vega and Cobb, 2023). Here too, TBI enhances these responses due to synergistic elevation of Ca^2+^, glutamate excitotoxicity, and oxidative injury.

Mitophagy plays a regulatory role in this cascade (Lu et al., 2023). Impaired mitophagy leads to retention of damaged mitochondria, sustained mtROS release, and subsequent inflammasome activation (Li et al., 2022). For instance, Parkin or PINK1 deficiency results in defective clearance of dysfunctional mitochondria and heightened NLRP3 activation via mtROS-dependent pathways (Tengesdal et al., 2023). Although much of this work derives from genetic or neurodegenerative models, traumatic injury produces similar disruptions in mitophagy, reinforcing this mechanism in TBI pathology.

In summary, mitochondrial ROS—especially overproduced or poorly scavenged mtROS—are now recognized as pivotal mediators linking MAM dysfunction to inflammation. Their dysregulation, particularly when MAM coupling is impaired, catalyzes inflammasome assembly (NLRP3, MAVS) and drives pathological inflammatory cascades. Mitophagy-related proteins (PINK1, Parkin) serve as essential counterbalances, preventing persistent mtROS accumulation and excessive inflammatory amplification (Luo et al., 2024; Su et al., 2023). Dysregulation of these MAM–mtROS–inflammasome interactions represents a central axis of secondary injury in TBI.

### 2.4 Regulation of apoptosis and autophagy signals

Beyond immediate Ca^2+^-mediated injury, MAM-regulated signaling exerts control over cell death programs such as autophagy and apoptosis. In cardiomyocytes subjected to ischemia–reperfusion, MAM-associated proteins such as Phosphofurin Acidic Cluster Sorting Protein 2 (PACS2) and FUN14 Domain-Containing Protein 1 (FUNDC1) have been shown to be vital: PACS2 maintains ER–mitochondrial juxtaposition, while FUNDC1 promotes MAM formation through tethering, preserving inter-organelle communication necessary for metabolic adaptation or injury responses (Chen et al., 2023). Although these findings originate largely from non-TBI models, similar alterations in PACS2 and FUNDC1 expression have been observed after traumatic brain injury, suggesting conserved regulation of MAM structural integrity during cellular stress.

Additionally, modulators such as Glycogen Synthase Kinase 3 Beta (GSK3β) and the Sigma-1 receptor (Sigma1R) at MAMs counteract calcium overload and ER stress, conferring cytoprotection during reperfusion injury (Wiseman et al., 2022). In the context of TBI, where ER stress, Ca^2+^ dysregulation, and oxidative stress converge rapidly after trauma, Sigma1R and GSK3β modulation at MAMs likely influences mitochondrial resilience and limits downstream apoptotic cascades. Preclinical TBI studies demonstrate that Sigma1R agonism stabilizes ER–mitochondria contacts, reduces ROS-driven mitochondrial damage, and attenuates CHOP-mediated apoptosis, reinforcing the relevance of this mechanism in traumatic injury.

Other MAM proteins—including Chloride Intracellular Channel 4 (CLIC4), NADPH Oxidase 4 (NOX4), and ATPase Family AAA Domain-Containing Protein 3A (ATAD3A)—are newly recognized regulators implicated in fine-tuning MAM structure and stress-response dynamics (Merighi and Lossi, 2022). Notably, NOX4 accumulation at MAMs increases localized ROS generation, which can sensitize the mPTP to opening and accelerate mitochondrial-mediated apoptosis. ATAD3A has been linked to altered mitochondrial dynamics and impaired mitophagy in models of neural stress—findings increasingly paralleled in TBI-associated mitochondrial fragmentation and defective organelle turnover.

These MAM-resident adaptors and enzymes integrate stress, metabolic, and redox cues, coordinating autophagic flux, mitochondrial integrity, and apoptotic signaling in response to cellular stressors (Zhang et al., 2025). Collectively, this protein network establishes MAMs as strategic control centers for balancing pro-survival autophagy and apoptotic pathways, determining cell fate under conditions of ER stress and oxidative challenge (Chen et al., 2024). In TBI specifically, where Ca^2+^ overload, ER stress activation, and mitochondrial dysfunction occur within minutes of injury, MAM remodeling likely determines the tipping point between adaptive mitophagy and catastrophic apoptosis—an emerging concept supported by recent TBI transcriptomic and ultrastructural analyses.

## 3 The association between stress crosstalk and neurological dysfunction

**Figure 3:**
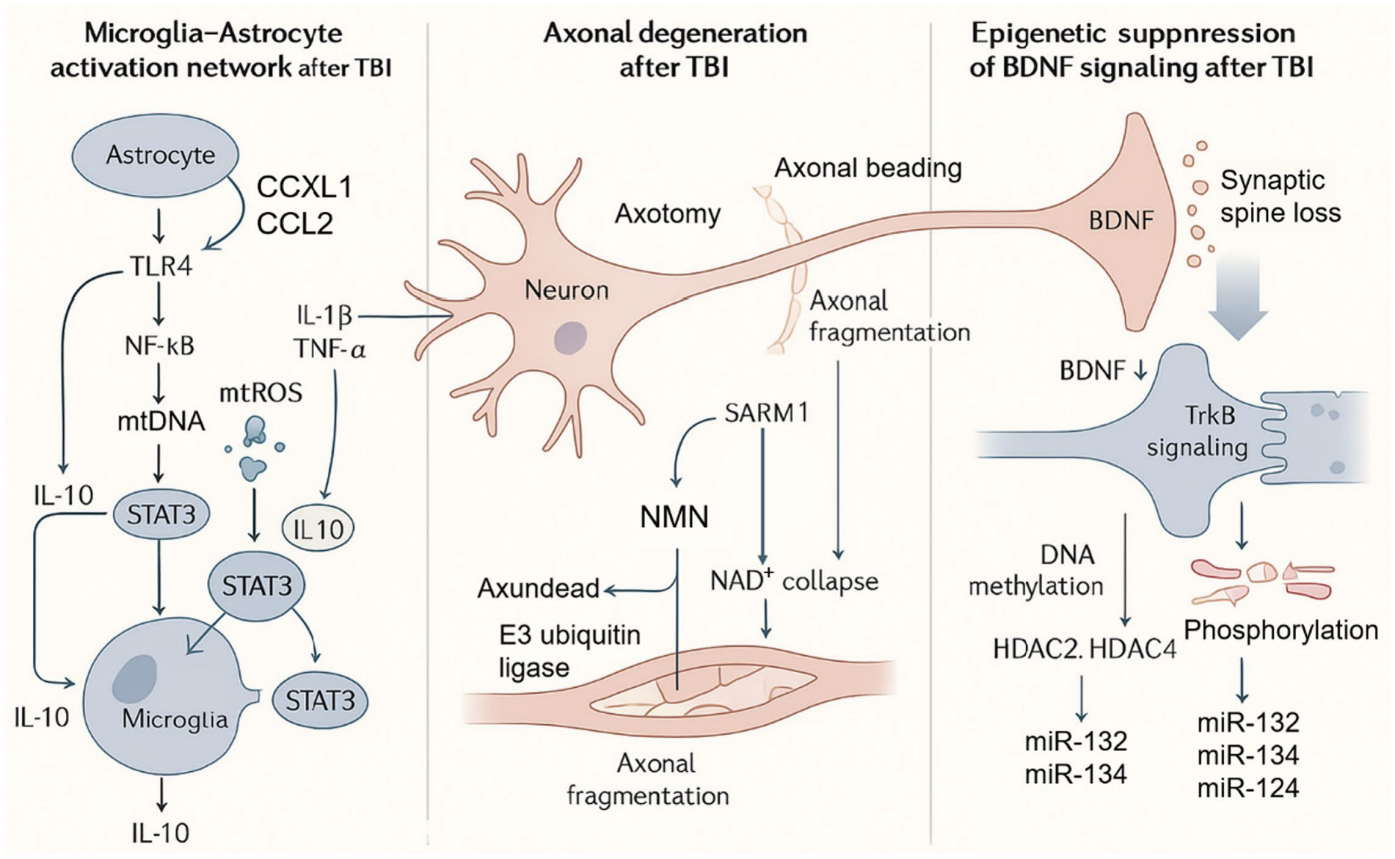
The association between stress crosstalk and neurological dysfunction. TBI, traumatic brain injury; TLR4, Toll-like receptor 4; NF-κB, nuclear factor kappa-light-chain-enhancer of activated B cells; IL-1β, interleukin-1 beta; TNF-*α*, tumor necrosis factor alpha; IL-10, interleukin-10; STAT3, signal transducer and activator of transcription 3; mtROS, mitochondrial reactive oxygen species; mtDNA, mitochondrial DNA; BDNF, brain-derived neurotrophic factor; TrkB, tropomyosin receptor kinase B; HDAC2/HDAC4, histone deacetylase 2 and 4; miR-132, microRNA-132; miR-134, microRNA-134; miR-124, microRNA-124; SARM1, sterile alpha and TIR motif-containing protein 1; NMN, nicotinamide mononucleotide; NAD^+^, nicotinamide adenine dinucleotide; E3, E3 ubiquitin ligase.

Lately, research on the pathogenesis of neurological disorders has increasingly focused on the interplay between stress signaling and neuronal dysfunction. Cellular stress triggers immune cell activation and the release of inflammatory cytokines and induces neuronal death and axonal injury through mitochondrial dysfunction. These processes further affect dendritic spine density, synaptic plasticity, and epigenetic regulation, ultimately leading to cognitive decline and behavioral abnormalities (Figure 3).

### 3.1 Neuroinflammation and glial activation

Traumatic brain injury triggers a robust neuroinflammatory response, primarily mediated by microglia and astrocytes, which act as the central effectors of immune signaling in the brain (Delpech et al., 2024). Upon activation, microglia transition from a surveillant to a pro-inflammatory phenotype (M1), releasing cytokines such as IL-1β, IL-6, and TNF-*α* (Bras et al., 2020). In TBI, this shift occurs within minutes to hours after injury and is tightly coupled to early metabolic and Ca^2+^ disturbances, making microglial reactivity a major early driver of secondary pathology.

These cytokines initiate a secondary injury cascade, amplifying oxidative and nitrosative stress, compromising neuronal integrity, and exacerbating blood–brain barrier disruption (Recasens et al., 2021). The transcription factor NF-κB acts as a master regulator of this inflammatory network, orchestrating gene expression programs for IL-1β, IL-6, and TNF-α, as well as the enzymes inducible nitric oxide synthase (iNOS) and cyclooxygenase-2 (COX-2) (Worthen et al., 2020). In TBI, NF-κB activation is enhanced by mitochondrial ROS, Ca^2+^ efflux from the ER, and mechanical membrane perturbations, integrating mechanical and biochemical injury signals.

In parallel, astrocytes undergo reactive transformation, characterized by increased GFAP expression and secretion of inflammatory mediators. They amplify microglial activation via NF-κB–dependent signaling and toll-like receptor 4 (TLR4) pathways, sustaining chronic neuroinflammation (Liu et al., 2025). Upstream of NF-κB, TLR4–MyD88 complex formation on glial membranes recruits IRAK and TRAF6, leading to IκB degradation and nuclear translocation of NF-κB, thus activating downstream pro-inflammatory genes (Zielinski, 2023). This TLR4-dependent mechanism is strongly implicated in TBI because DAMPs (HMGB1, ATP, extracellular mtDNA) are abundantly released from mechanically injured neurons.

Meanwhile, NLRP3 inflammasome activation within microglia serves as another critical node in this cascade. Mitochondrial damage, ROS accumulation, and Ca^2+^ dysregulation converge to trigger NLRP3 oligomerization, ASC recruitment, and caspase-1 activation, resulting in maturation and release of IL-1β (Xu et al., 2023). The convergence of these triggers occurs at ER–mitochondria contact sites (MAMs), which function as structural platforms for integrating Ca^2+^ flux, mtROS production, and inflammasome assembly—mechanistic features increasingly recognized in TBI models.

Conversely, IL-10, a canonical anti-inflammatory cytokine, exerts negative feedback control over microglial activation. Through Signal Transducer and Activator of Transcription 3 (STAT3) phosphorylation, IL-10 suppresses NF-κB signaling and downregulates pro-inflammatory gene transcription, promoting a transition toward an M2 reparative phenotype (Satoh et al., 2025). TBI severity and temporal phase influence the IL-10/STAT3 axis, and insufficient activation of this pathway has been associated with prolonged microglial reactivity and chronic neuroinflammation in both experimental and clinical TBI studies.

The balance between IL-1β/NF-κB–driven inflammation and IL-10/STAT3-mediated resolution determines glial functional polarization and the outcome of neuroinflammatory injury (Dogan et al., 2023). Collectively, these findings underscore that NF-κB, NLRP3, and STAT3 represent pivotal regulatory hubs linking cytokine signaling with microglial and astrocytic reactivity, thereby shaping the inflammatory milieu following TBI (Huang et al., 2024). Moreover, their activation is closely intertwined with mitochondrial dysfunction and MAM-mediated Ca^2+^/ROS signaling, positioning inflammatory stress as both a downstream effector and a potent amplifier of early metabolic injury in TBI.

### 3.2 Axonal degeneration and SARM1-mediated pathways

Axonal degeneration is a hallmark of TBI, representing one of the earliest and most irreversible cellular events following mechanical insult (Nemeth et al., 2024). The execution of programmed axon degeneration is distinct from apoptosis and is orchestrated by a molecular network centered around Sterile Alpha and TIR Motif-Containing Protein 1 (SARM1) (Wei et al., 2022). In TBI, this pathway is rapidly engaged due to diffuse axonal stretch, membrane rupture, and early metabolic crisis, making the SARM1 axis a critical contributor to secondary injury progression.

Under physiological conditions, axonal survival is maintained by Nicotinamide Mononucleotide Adenylyltransferase 2 (NMNAT2), which sustains NAD^+^ homeostasis and metabolic competence along axons (Lee et al., 2024). Upon axonal injury, NMNAT2 is rapidly degraded, leading to accumulation of its substrate nicotinamide mononucleotide (NMN) and depletion of NAD^+^. This metabolic shift directly activates SARM1, whose TIR domain possesses intrinsic NADase activity, catalyzing NAD^+^ breakdown and triggering catastrophic energetic collapse (Liu et al., 2024). In TBI models, the speed of NMNAT2 depletion is accelerated by Ca^2+^ influx and microtubule transport failure, reflecting the combined impact of mechanical stress and metabolic dysfunction.

Activated SARM1 drives rapid consumption of NAD^+^, resulting in ATP depletion, ionic imbalance, and activation of downstream Ca^2+^-dependent proteases such as Calpain-2, which degrade cytoskeletal components including neurofilaments and microtubules (Cruceriu et al., 2020). This biochemical cascade culminates in axonal fragmentation and disassembly of microtubule networks. These events align closely with diffuse axonal injury pathology in TBI patients, where calpain-mediated spectrin breakdown products and microtubule collapse are early histopathological markers.

Parallel signaling pathways intersect with SARM1-mediated degeneration. The dual leucine zipper kinase (DLK)–c-Jun N-terminal kinase (JNK) axis is upregulated in injured axons, promoting phosphorylation of c-Jun and activation of stress-responsive gene transcription that facilitates axonal dismantling (York et al., 2024). Although extensively studied in developmental and toxic neuropathy models, TBI studies similarly show DLK–JNK activation in damaged white matter tracts, linking mechanical injury to transcriptional stress signaling. In addition, Axundead, a recently characterized E3 ubiquitin ligase, acts upstream to regulate SARM1 activation, controlling the threshold for NAD^+^ consumption and axonal self-destruction (Wang et al., 2023).

Further downstream, caspase-3 activation contributes to secondary axonal degeneration, coordinating with Calpain-2–mediated proteolysis under conditions of severe energy crisis (Xie et al., 2024). These mechanisms converge on shared metabolic failure, highlighting how mitochondrial dysfunction, Ca^2+^ overload, and ATP depletion reinforce the SARM1 degeneration pathway.

Collectively, these findings define a metabolic–enzymatic cascade wherein NMNAT2 loss, NMN accumulation, and SARM1 activation serve as the initiating events of Wallerian-like degeneration following traumatic insult. The SARM1–NADase axis, together with DLK–JNK signaling and Calpain-2–caspase protease systems, constitutes the molecular framework that governs axonal self-destruction (Lee et al., 2023). Emerging evidence suggests that SARM1-mediated degeneration is amplified by mitochondrial Ca^2+^ dysregulation and mtROS production—both of which are influenced by ER–mitochondria contact (MAM) remodeling after TBI—placing SARM1 at the intersection of mechanical and metabolic secondary injury mechanisms.

### 3.3 Cognitive impairment, epigenetic regulation, and BDNF signaling

Traumatic brain injury (TBI) is frequently accompanied by cognitive and memory dysfunction, which stems from synaptic plasticity impairment, neuronal loss, and dysregulated neurotrophic signaling within the hippocampus and prefrontal cortex (Fakhri et al., 2022). These deficits are particularly pronounced in moderate-to-severe TBI, where early metabolic collapse, excitotoxicity, and neuroinflammation converge to disrupt synaptic homeostasis.

Among neurotrophic factors, brain-derived neurotrophic factor (BDNF) plays a pivotal role in supporting neuronal survival, axonal growth, and synaptic remodeling. BDNF exerts its effects primarily through binding to its high-affinity receptor TrkB, triggering downstream cascades such as PI3K–Akt, MAPK–ERK, and PLCγ pathways, which converge on transcriptional activation of plasticity-related genes (Lindholm et al., 2006). In TBI, these signaling cascades are further disrupted by mitochondrial dysfunction and Ca^2+^ dysregulation, which impair activity-dependent BDNF release and synaptic responsiveness.

Post-TBI, the expression of BDNF and TrkB signaling is markedly reduced, leading to diminished activation of CREB—a transcription factor essential for long-term potentiation (LTP) and memory consolidation (Jeon et al., 2023). Reduced BDNF–CREB signaling correlates with impaired synaptic transmission and spatial learning deficits observed in experimental models (Patel et al., 2023). These effects are often exacerbated by ROS accumulation, ER stress activation, and MAM remodeling, all of which negatively influence CREB phosphorylation and synaptic plasticity.

Emerging evidence indicates that epigenetic dysregulation significantly contributes to these alterations. Increased DNA methylation within the BDNF promoter region suppresses its transcription in the injured brain (Hafycz et al., 2023). Similarly, aberrant histone modifications, including deacetylation of histone H3 and methylation of H3K9, lead to chromatin condensation and reduced transcriptional accessibility of neuroplasticity-associated genes (Zyryanova et al., 2021). Although these epigenetic mechanisms are supported by broader neurodegeneration and stress-model literature, TBI studies increasingly reproduce these findings, reinforcing their relevance in trauma-induced cognitive decline.

The histone deacetylases (HDACs), particularly HDAC2 and HDAC4, are upregulated after TBI, promoting transcriptional repression of BDNF, synapsin I, and other neuronal survival genes (Karvandi et al., 2023). Pharmacological inhibition or genetic silencing of HDACs restores histone acetylation, BDNF expression, and cognitive performance in experimental models (Surico et al., 2025). This aligns with observations that HDAC inhibition also modulates mitochondrial dynamics and inflammatory pathways, suggesting multi-level benefits in TBI.

MicroRNAs (miRNAs) also participate in the epigenetic regulation of post-TBI synaptic plasticity. For instance, miR-132 and miR-134—regulators of mRNAs encoding synaptic scaffolding proteins and CREB modulators—are significantly altered after injury (Hao et al., 2022). Dysregulation of these miRNAs leads to destabilization of dendritic spines and attenuation of synaptic signaling (Losurdo et al., 2020). These miRNAs are also responsive to oxidative and inflammatory cues, linking epigenetic disruption with early metabolic stress following TBI.

Moreover, miR-124—a neuron-enriched microRNA—exhibits decreased expression following TBI, resulting in activation of pro-inflammatory transcription factors and suppression of neuronal differentiation pathways (Tan et al., 2024). Restoration of miR-124 expression alleviates neuroinflammation, enhances BDNF–TrkB signaling, and improves cognitive outcomes (Vahab et al., 2025). These observations help bridge neuroinflammation (Section 3.1) with neuroplasticity deficits.

Collectively, these findings delineate a multi-layered regulatory network in which epigenetic modifications (DNA methylation, histone acetylation, miRNA interference) converge on BDNF–TrkB–CREB signaling. Disruption of this network underlies synaptic dysfunction and cognitive impairment after TBI (Li et al., 2025). Importantly, this network is highly sensitive to upstream mitochondrial injury, Ca^2+^ dysregulation, and MAM remodeling, situating epigenetic and neurotrophic disturbances as downstream manifestations of the early metabolic crisis induced by TBI.

## 4 Therapeutic strategies and intervention progress

**Figure 4:**
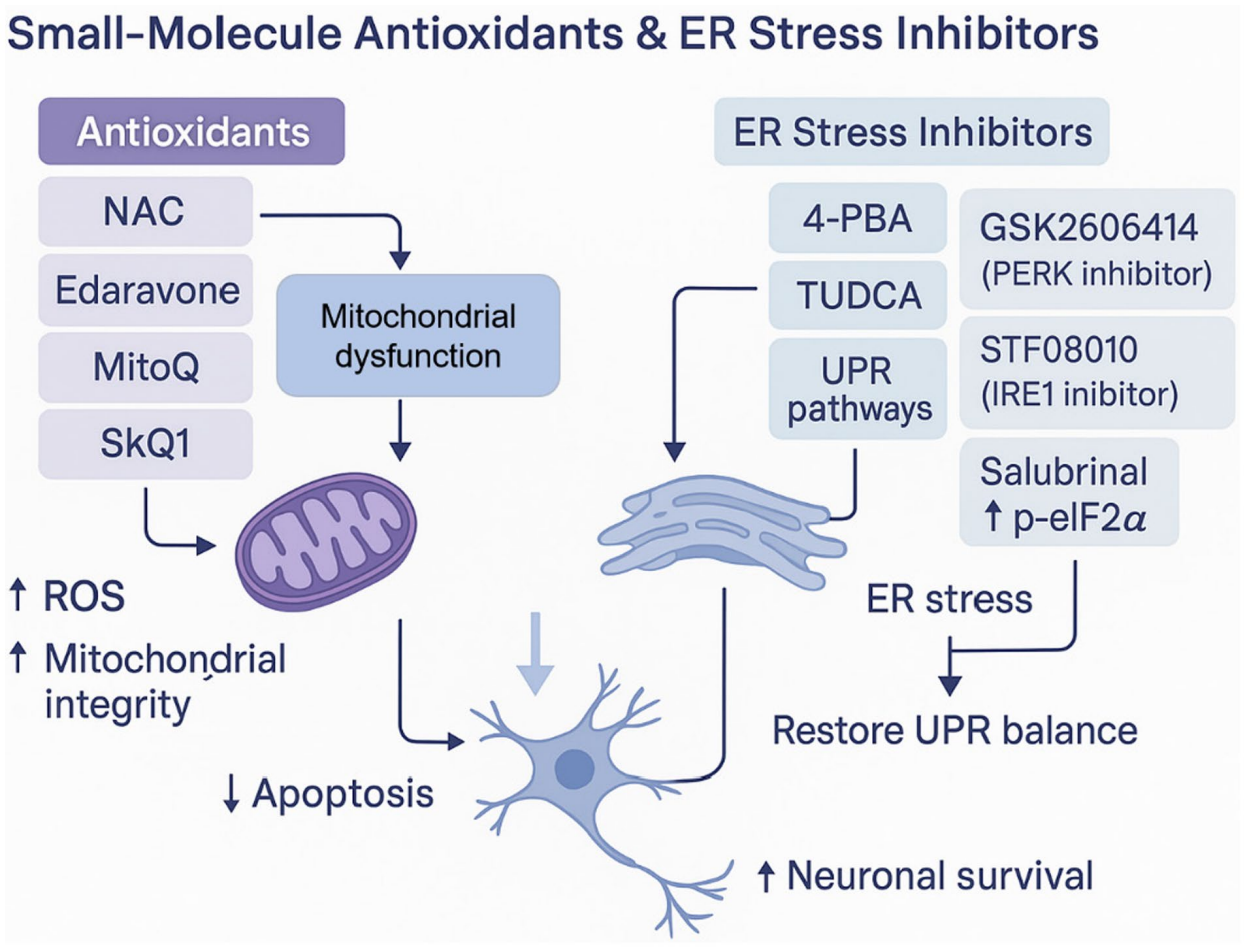
Therapeutic strategies and intervention progress. ROS, reactive oxygen species; Mitochondrial dysfunction, mitochondrial dysfunction; ER stress, endoplasmic reticulum stress; UPR, unfolded protein response; PERK, protein kinase RNA-like endoplasmic reticulum kinase; IRE1α, inositol-requiring enzyme 1 alpha; eIF2α, eukaryotic initiation factor 2 alpha; p-eIF2α, phosphorylated eIF2α.

With the growing understanding of the pathogenesis of neurodegenerative diseases, increasing research efforts have been devoted to exploring novel strategies to reverse neuronal apoptosis. Oxidative stress and mitochondrial dysfunction are key contributors to neuronal damage, making small-molecule drugs—such as antioxidants and ER stress inhibitors (e.g., NAC, vitamin E, TUDCA)—a major focus in therapeutic development. In addition, biologics including stem cells, exosomes, and siRNA have shown significant potential in repairing neuronal injury[150]Meanwhile, emerging approaches such as nanodrug delivery systems and combination therapies offer more targeted and efficient means to intervene in neuronal apoptosis (Figure 4).

### 4.1 Small-molecule antioxidants and ER stress inhibitors

Therapeutic strategies targeting oxidative stress and endoplasmic reticulum stress (ERS) have attracted increasing attention due to their central involvement in secondary neuronal injury after TBI (Li et al., 2023). Among antioxidant compounds, NAC is one of the most extensively studied. Acting as a precursor of glutathione (GSH), NAC replenishes intracellular antioxidant capacity, scavenges ROS, and maintains redox homeostasis in injured neurons (Delpech et al., 2024). In experimental TBI models, NAC administration attenuates lipid peroxidation, DNA oxidation, and mitochondrial dysfunction, thereby improving neuronal survival and cognitive recovery (Lin and Qi, 2023). However, NAC’s therapeutic window is narrow, and its efficacy decreases when administered beyond early post-injury phases, underscoring the importance of timing in clinical translation.

Edaravone, a clinically approved free radical scavenger, neutralizes hydroxyl radicals and inhibits lipid peroxidation chain reactions, protecting cell membranes from oxidative injury (Cheng et al., 2022). By stabilizing mitochondrial integrity and suppressing cytochrome c release, Edaravone reduces apoptotic signaling and inflammatory amplification after TBI (Simpson and Oliver, 2020). Despite promising effects, variable BBB penetration and the need for repeated high dosing remain challenges for widespread clinical use in TBI.

MitoQ, a mitochondria-targeted antioxidant, delivers ubiquinone moieties directly to the mitochondrial matrix via a triphenylphosphonium (TPP^+^) carrier. MitoQ effectively prevents mtROS accumulation, maintains ATP synthesis, and inhibits NF-κB–mediated inflammatory gene expression (Mursaleen et al., 2023). Similarly, SkQ1, another mitochondria-directed antioxidant, demonstrates robust efficacy in reducing ROS-induced neuronal death by preserving mitochondrial bioenergetics (MacDougall et al., 2021). Nevertheless, both MitoQ and SkQ1 remain largely preclinical, and concerns about TPP^+^-dependent distribution, long-term safety, and dose-dependent pro-oxidant effects have limited their movement into TBI clinical trials.

In addition to antioxidative therapy, ER stress inhibitors have emerged as promising interventions to restore proteostasis and UPR balance following trauma (van der Worp et al., 2010). 4-PBA, a chemical chaperone, alleviates protein misfolding, suppresses PERK–CHOP–eIF2*α* pathway activation, and mitigates apoptosis in stressed neurons (Lange and Inal, 2023). Similarly, TUDCA reduces ER stress by stabilizing protein conformation and enhancing ER–mitochondria communication, thereby preventing Ca^2+^ overload and oxidative damage (Egan et al., 2018). However, both agents exert broad actions across multiple stress pathways, and their optimal dosing, specificity, and long-term impact on physiological UPR remain areas of concern for clinical translation.

Moreover, small-molecule inhibitors targeting specific branches of the UPR show neuroprotective potential. For example, GSK2606414, a selective PERK inhibitor, downregulates CHOP induction and decreases neuronal apoptosis, while STF083010, an IRE1α endonuclease inhibitor, suppresses XBP1 splicing and ER-derived inflammatory signals (Bellotti et al., 2024). Salubrinal, which enhances eIF2α phosphorylation, protects against ER stress-induced apoptosis by reducing CHOP expression and restoring ER function (Hampel et al., 2021). Despite these benefits, PERK/IRE1 inhibition may compromise essential adaptive UPR functions or heighten vulnerability to infections, and most data stem from non-TBI models, requiring cautious interpretation.

These findings collectively suggest that antioxidants (NAC, Edaravone, MitoQ, SkQ1) and ERS inhibitors (4-PBA, TUDCA, GSK2606414, STF083010, Salubrinal) act through complementary mechanisms to reduce ROS, stabilize protein folding, and restore ER–mitochondria homeostasis (Blennow and Zetterberg, 2018). Such combination approaches targeting oxidative and ER stress axes may represent a synergistic therapeutic paradigm for mitigating neuronal death and promoting functional recovery after TBI (Palmqvist et al., 2020). Still, clinical translation remains limited due to patient heterogeneity, narrow therapeutic windows, and insufficient large-scale trials. Future efforts should incorporate biomarker-guided dosing and timing to align redox and ER interventions with individual stress-response profiles.

### 4.2 Biologics and recombinant therapeutics

Beyond small-molecule interventions, biologic agents have emerged as promising therapeutics for targeting complex injury cascades following TBI (Khalil et al., 2024). Among neurotrophic factors, nerve growth factor (NGF) and BDNF are the most extensively investigated. Exogenous administration of NGF promotes neuronal survival, axon regeneration, and synaptic remodeling, while BDNF enhances plasticity and LTP through activation of TrkA and TrkB receptors, respectively (McGettigan et al., 2023). BDNF–TrkB signaling stimulates PI3K–Akt, MAPK–ERK, and PLCγ pathways, facilitating neuronal survival and synaptogenesis after injury. Importantly, TBI-induced mitochondrial dysfunction and chronic inflammation can suppress endogenous BDNF production, making neurotrophin replacement a potentially valuable—yet timing-dependent—strategy.

Reduced endogenous BDNF expression post-TBI can be compensated by recombinant BDNF protein delivery or gene therapy vectors, which restore signaling and cognitive function (Hinderer et al., 2018). However, recombinant neurotrophins typically show short half-lives, limited BBB penetration, and dose-dependent adverse effects, and many supportive data originate from non-TBI neurodegeneration models, underscoring the need for cautious interpretation in the TBI context.

In addition to direct neurotrophin supplementation, stem cell-derived biologics—particularly mesenchymal stem cell (MSC)-derived exosomes—exert neuroprotective effects via paracrine signaling. These extracellular vesicles carry miRNAs, proteins, and lipids that modulate microglial polarization, reduce inflammation, and enhance axonal regeneration (Wang et al., 2019). Exosomal miR-124 and miR-21, delivered via MSC exosomes, have been shown to suppress NF-κB signaling and augment BDNF expression, promoting neuronal differentiation and synaptic recovery (Ngo et al., 2022). Although exosome biology has been extensively characterized in stroke and neurodegenerative models, TBI studies increasingly confirm their ability to modulate neuroinflammation and rebuild synaptic networks.

Similarly, neural stem cell (NSC)-derived exosomes containing miR-9 and miR-219 facilitate remyelination and synaptic network reconstruction after TBI (Pan et al., 2025). Yet, variability in exosome isolation, scalability, and delivery routes continues to impede translation into standardized clinical therapy.

Recombinant protein therapies, such as erythropoietin (EPO), also demonstrate pleiotropic neuroprotective properties beyond hematopoiesis. EPO reduces oxidative stress, inflammation, and apoptosis by activating JAK2–STAT5 and PI3K–Akt pathways, thereby promoting cell survival and angiogenesis (He et al., 2024). Nevertheless, clinical trials of EPO in TBI have yielded mixed outcomes, partly due to dosing challenges and thrombotic risks, highlighting the need for biomarker-guided administration.

Furthermore, monoclonal antibodies (mAbs) targeting pro-inflammatory mediators—such as anti-TNF-α or anti-IL-1β antibodies—effectively attenuate neuroinflammation, reduce microglial activation, and preserve neuronal integrity (Chaplin et al., 2020). Still, systemic immune suppression and limited BBB permeability remain major barriers for chronic or repeated use of cytokine-neutralizing antibodies in TBI patients.

Collectively, biologic therapies—including neurotrophins, stem cell–derived exosomes, recombinant proteins, and cytokine-targeting antibodies—offer multi-targeted approaches that integrate anti-inflammatory, anti-apoptotic, and neuroregenerative mechanisms, providing a complementary strategy to small-molecule drugs for post-traumatic repair (Lotfy et al., 2023). However, their translation to clinical practice requires addressing challenges such as delivery optimization, safety monitoring, pharmacokinetics, and the heterogeneity of TBI pathology.

### 4.3 Emerging technologies and combination therapies

Recent advances in nanotechnology, biomaterials, and drug-delivery platforms have provided new opportunities for precise intervention in TBI-related secondary injury cascades (Zuin et al., 2022). Nanocarrier-based systems—such as liposomes, polymeric nanoparticles, solid-lipid nanoparticles, and metal–organic frameworks (MOFs)—enable controlled release, targeted delivery, and improved bioavailability of therapeutic agents across the BBB (Perneczky and Froelich, 2025). These systems can encapsulate small-molecule antioxidants, peptides, or nucleic acids, ensuring sustained protection against oxidative stress and neuroinflammation (Erickson et al., 2025). PEGylated liposomes and PLGA-based nanoparticles, for example, have been engineered to enhance brain penetration and prolong circulatory half-life, thereby reducing systemic toxicity and enhancing TBI-specific accumulation (Daniore et al., 2024). In addition, surface modification of nanocarriers with ligands, antibodies, or cell-penetrating peptides (CPPs) allows receptor-mediated transcytosis through endothelial cells, improving selective delivery to injured brain regions (Lee et al., 2022).

Exosome-mimetic nanoparticles represent another promising platform, combining the biocompatibility of natural vesicles with the customizability of synthetic carriers. These systems have been designed to co-deliver neurotrophic factors and anti-inflammatory molecules, achieving synergistic neuroprotection (Olaghere et al., 2025). Although much of the design framework originates from oncology and stroke models, emerging TBI studies support their ability to modulate glial activation, reduce mtROS accumulation, and enhance axonal repair, suggesting cross-platform translational relevance.

Beyond single-agent therapy, combination approaches targeting multiple injury pathways—including oxidative stress, ER stress, inflammation, and apoptosis—have demonstrated enhanced efficacy. Co-delivery of antioxidants with UPR modulators or anti-inflammatory biologics can simultaneously restore redox balance and proteostasis, improving neuronal resilience (Cai et al., 2024). For example, nanoparticle-mediated co-delivery of MitoQ with 4-PBA has been shown to attenuate mitochondrial oxidative damage while suppressing CHOP-dependent apoptosis (Velmurugan et al., 2024), illustrating the advantage of dual-target mechanistic integration in TBI models.

Gene–drug hybrid delivery systems, incorporating siRNA, miRNA mimics, or CRISPR/Cas9 components within nanocarriers, enable spatiotemporal regulation of key signaling pathways. These next-generation technologies allow targeted silencing of pro-apoptotic genes or activation of neuroprotective transcriptional programs, enhancing therapeutic precision (Martins et al., 2023). However, concerns remain regarding off-target editing, immune responses to CRISPR components, and heterogeneous uptake across injured brain regions—factors that complicate their clinical translation in TBI.

Emerging stimuli-responsive nanoplatforms, which release cargo in response to pH, ROS, or enzyme triggers, further refine drug localization and dosing control within heterogeneous post-traumatic microenvironments (Eteleeb et al., 2024). Still, differences between rodent and human BBB properties, long-term nanomaterial biodegradation, and safety concerns limit the direct extrapolation of many of these findings to clinical TBI.

Collectively, these technological innovations—including BBB-penetrant nanocarriers, biomimetic vesicles, and multifunctional combination regimens—offer transformative potential for overcoming current limitations of neuroprotective therapies, advancing toward personalized and integrative treatment strategies for TBI (Sun et al., 2023).

Nonetheless, optimization of delivery routes, validation in large-animal TBI models, and rigorous safety profiling remain crucial steps before these approaches can progress toward clinical translation.

## 5 Clinical transformation and challenges

**Figure 5:**
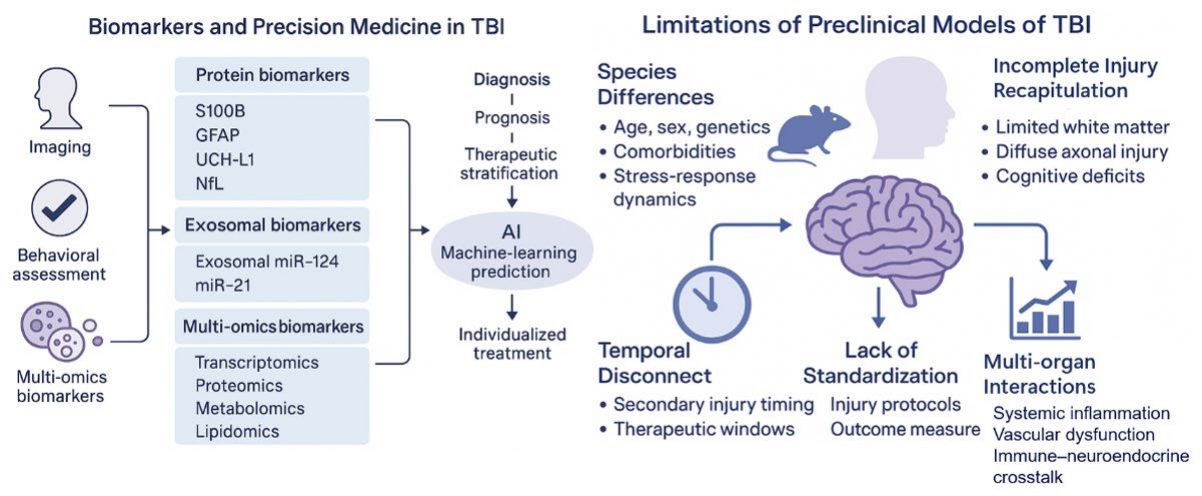
Clinical transformation and challenges. TBI, traumatic brain injury; S100B, S100 calcium-binding protein B; GFAP, glial fibrillary acidic protein; UCH-L1, ubiquitin C-terminal hydrolase L1; NfL, neurofilament light chain; miR-124, microRNA-124; miR-21, microRNA-21; AI, artificial intelligence.

During the process of translating basic research into clinical applications, several key challenges emerge. Firstly, there are significant differences between animal models and human patients in terms of pathological mechanisms, immune responses, and comorbidities, which limit the translational relevance of preclinical findings. Secondly, biomarkers used for personalized therapy often suffer from limited sensitivity and are influenced by substantial inter-individual variability, making accurate patient stratification and therapeutic prediction difficult. Additionally, clinical implementation of new therapies faces risks such as immune-related adverse effects and a lack of effective tools for long-term efficacy monitoring. These factors overall hinder the widespread application of research outcomes in clinical settings, highlighting the urgent need to optimize model systems, improve diagnostic technologies, and establish comprehensive safety assessment frameworks (Figure 5).

### 5.1 Experimental models and translational gaps

Although significant mechanistic progress has been made using preclinical models, translating laboratory findings into effective clinical therapies for TBI remains a major challenge (Huang et al., 2023). Most current studies rely on rodent models such as CCI, fluid percussion injury (FPI), and weight-drop models, which successfully reproduce key biomechanical and histopathological features of human TBI and provide opportunities to investigate cerebrospinal fluid (CSF) biomarkers associated with neural injury (Kress et al., 2023). However, these models incompletely capture the heterogeneity of human TBI—especially variability in age, sex, comorbidities, genetic background, and injury biomechanics—all of which strongly influence stress-response dynamics and treatment efficacy (Zarini-Gakiye et al., 2021).

Moreover, the temporal dynamics of secondary injury differ markedly between species. Rodents exhibit accelerated metabolic recovery, faster resolution of mitochondrial dysfunction, and enhanced neuroplasticity relative to humans, leading to mismatches in optimal therapeutic windows for targeting oxidative stress, ER stress, and inflammatory cascades (Fedoruk et al., 2023). As a result, neuroprotective compounds effective in rodents frequently fail to show efficacy in clinical trials (Golovachev et al., 2024), highlighting the biological and temporal disconnect between preclinical stress-pathway modulation and human TBI progression.

A further limitation lies in the scale and organization of rodent brains. The absence of a gyrencephalic cortex, reduced white-matter volume, and simpler connectivity patterns restrict modeling of diffuse axonal injury, higher-order cognitive deficits, and long-range network dysfunction—core clinical features of moderate-to-severe human TBI (Fesharaki-Zadeh, 2022). Although large-animal models (e.g., pigs, non-human primates) better replicate human neuroanatomy, axonal tract architecture, and biomechanical loading properties, their use is limited by ethical, financial, and logistical constraints (Ionescu et al., 2025).

Additionally, variability in injury induction protocols, outcome measures, and endpoint selection across laboratories reduces reproducibility and comparability of findings (Li et al., 2025). Standardization of model severity, evaluation timelines, stress-response readouts (e.g., mtROS, MAM remodeling, inflammasome activation), and behavioral assessments is urgently needed to enhance cross-study validity. The failure to integrate multi-organ interactions—such as systemic inflammation, vascular dysfunction, and immune–neuroendocrine crosstalk—further contributes to the translational gap (Wang et al., 2023). Clinically, TBI is a multisystem disorder, yet current preclinical models primarily focus on isolated neural injury, failing to reflect interactions between peripheral inflammation and central stress pathways.

Addressing these limitations will require multi-scale modeling, humanized systems, and organoid-based platforms capable of recapitulating human-specific features of ER–mitochondria communication, Ca^2+^ dysregulation, and neuroimmune activation (Zhou and Glass, 2025). Together, these factors underscore the need for integrative experimental designs and standardized translational frameworks that bridge the divide between bench and bedside in TBI research (He et al., 2023).

### 5.2 Biomarkers and precision subtyping

Identification of reliable biomarkers for diagnosis, prognosis, and therapeutic stratification represents a central goal in advancing precision medicine for TBI (Zhuang, 2025). Current clinical evaluation largely relies on neuroimaging and behavioral assessment, which lack molecular specificity and often fail to detect subclinical injuries or individual variability in recovery trajectories (Hampel et al., 2023). Therefore, molecular biomarkers reflecting cellular stress, neuroinflammation, and metabolic dysfunction are increasingly investigated as objective indicators of injury severity and outcome (Rossi et al., 2023).

Among candidate markers, S100B, glial fibrillary acidic protein (GFAP), and ubiquitin C-terminal hydrolase L1 (UCH-L1) have been validated as indicators of astroglial and neuronal injury (Marei et al., 2023). Elevated neurofilament light chain (NfL) levels in cerebrospinal fluid and plasma correlate with axonal damage and cognitive decline, providing a quantifiable measure of neurodegeneration (Hurley et al., 2023). Importantly, these markers also mirror key stress processes—including cytoskeletal breakdown, mitochondrial dysfunction, and persistent neuroinflammation—linking biomarker fluctuations to the mechanistic cascades described in earlier sections.

Emerging evidence supports the use of extracellular vesicles (EVs) and exosomal cargo as minimally invasive biomarkers. EVs isolated from blood or CSF contain miRNAs, proteins, and lipid metabolites reflective of the cellular origin and injury context (Hernandez et al., 2024). For instance, exosomal miR-124 and miR-21 levels correlate with microglial activation and neuroinflammatory states, providing insight into ongoing stress signaling beyond what can be detected with traditional serum markers (de Rojas et al., 2021). Although many EV assays remain in early-stage development, TBI-specific alterations in exosomal miRNAs and mitochondrial proteins highlight their potential as dynamic reporters of intracellular stress pathways—particularly MAM remodeling, mtROS accumulation, and Ca^2+^ dysregulation.

Beyond individual molecules, multi-omics profiling—integrating transcriptomics, proteomics, metabolomics, and lipidomics—enables comprehensive mapping of TBI pathophysiology and identification of molecular subtypes (Bellou et al., 2025). This systems-level approach facilitates patient stratification, allowing targeted therapies tailored to distinct stress-response signatures (Ossenkoppele and van der Flier, 2023). For example, metabolomic indicators of mitochondrial dysfunction or proteomic signatures of ER stress may identify patient clusters more responsive to antioxidant or UPR-modulating treatments.

Moreover, combining biomarker panels with machine-learning algorithms enhances predictive accuracy, supporting early prognosis and dynamic monitoring of therapeutic efficacy (Foley and Wilcock, 2024). AI-driven models can identify hidden biomarker patterns that delineate subgroups with distinct outcomes or treatment responses (Zhao et al., 2022). However, challenges remain—including small cohort sizes, inter-assay variability, and lack of harmonized biomarker thresholds—that currently limit the clinical deployment of AI-assisted TBI diagnostics.

Implementing precision subtyping based on biomarker signatures will enable a shift from uniform interventions toward personalized therapeutic regimens, optimizing efficacy while minimizing adverse effects (Chen et al., 2024). Collectively, the integration of multi-modal biomarkers with AI-enabled analytics represents a transformative direction for achieving individualized diagnosis and treatment in TBI, though rigorous validation across diverse clinical populations remains essential (Tremblay-Mercier et al., 2021).

## 6 Future outlook

### 6.1 Multi-omics approaches uncovering mechanistic insights

Recent advances in high-throughput omics technologies have enabled a systems-level understanding of the complex molecular networks underlying TBI pathophysiology (Erickson et al., 2025). Integrating genomics, epigenomics, transcriptomics, proteomics, metabolomics, and lipidomics provides a comprehensive landscape of how cellular stress, inflammation, and neurodegeneration interact across spatial and temporal scales (Qi et al., 2025). Unlike single-biomarker approaches, multi-omics allows direct mapping of TBI-specific responses, including early metabolic crisis, Ca^2+^ dysregulation, and inflammatory amplification.

Transcriptomic profiling of injured brain regions reveals dynamic regulation of genes involved in oxidative stress, UPR activation, autophagy, and immune signaling, delineating distinct molecular phases of secondary injury (Reicher et al., 2025). These signatures align with experimentally defined stress pathways—such as PERK–CHOP activation, mitochondrial biogenesis suppression, and innate immune priming—demonstrating that transcriptional waves recapitulate core mechanisms of TBI pathology.

Proteomic and metabolomic analyses complement these findings by identifying perturbations in energy metabolism, amino acid turnover, and lipid peroxidation that mirror mitochondrial dysfunction and redox imbalance (Cruz Navarro et al., 2022). Particularly, alterations in TCA intermediates, acylcarnitines, and phospholipid metabolites reflect impaired ER–mitochondria communication and mtROS accumulation, directly linking omics signatures to MAM-associated stress processes.

By correlating multi-omic datasets, researchers can reconstruct causal networks linking gene-expression changes to protein interactions and metabolic flux alterations, uncovering cross-organellar communication mechanisms such as ER–mitochondria crosstalk (Peng et al., 2025). These integrative analyses have highlighted master regulators—including mitochondrial Ca^2+^ transporters, inflammasome components, and autophagy adaptors—as convergence points where transcriptional, proteomic, and metabolic disruptions intersect.

Moreover, integrative omics facilitates the discovery of novel therapeutic targets, enabling prioritization of stress-response regulators and signaling nodes for pharmacological intervention (Canal-Garcia et al., 2024). Examples include identification of mitochondrial redox enzymes, lipid-metabolizing proteins at MAMs, and regulators of microglial–neuronal communication as high-value targets in TBI. However, many omics-derived targets originate from rodent models or non-TBI neurological conditions, necessitating careful validation in human cohorts.

As computational frameworks and network-biology tools continue to evolve, multi-omics integration is expected to reveal hidden layers of regulation, guiding mechanism-based therapy design and biomarker discovery in TBI (Kim et al., 2023). Nevertheless, challenges such as small clinical sample sizes, heterogeneity of injury patterns, and batch effects remain obstacles to full clinical translation, highlighting the need for standardized pipelines and multicenter data harmonization.

### 6.2 Precision medicine and individualized therapeutic strategies

The emerging paradigm of precision medicine seeks to tailor interventions based on individual molecular signatures, genetic predispositions, and pathophysiological subtypes of TBI (Komori et al., 2022). Given the remarkable heterogeneity in injury severity, neuroinflammatory response, mitochondrial vulnerability, and repair capacity, one-size-fits-all treatment approaches often yield suboptimal outcomes (Kimura et al., 2025). TBI-specific variability in Ca^2+^ dysregulation, ER stress intensity, and mtROS-driven inflammatory cascades further underscores the need for individualized treatment models.

Integrating molecular profiling with clinical phenotyping enables stratification of patients into subgroups characterized by distinct stress-response patterns and therapeutic susceptibilities (Rakaee et al., 2025). For example, patients exhibiting dominant oxidative stress signatures might benefit more from mitochondria-targeted antioxidants, whereas those with pronounced ER stress or inflammasome activation may respond preferentially to UPR modulators or anti-inflammatory biologics. These stratified categories map directly onto the mechanistic pathways described earlier—MAM dysfunction, mitochondrial collapse, neuroinflammation—offering a biologically grounded framework for patient selection.

Personalized regimens may incorporate combinations of antioxidants, ERS inhibitors, biologics, or gene modulators selected according to each patient’s biochemical and genetic context (Li et al., 2022). Such strategies can be guided by biomarker panels identified through omics-driven classification, ensuring targeted engagement of dysregulated pathways (Li et al., 2025). However, many omics-derived stratification tools originate from small cohorts or non-TBI neurological datasets, requiring rigorous validation in larger and more diverse TBI populations.

Future research will benefit from adaptive trial designs, real-time biomarker monitoring, and dynamic dosing algorithms, allowing iterative refinement of interventions to match evolving injury states (Amato et al., 2025). These adaptive frameworks are particularly suited for TBI, where secondary injury evolves rapidly and therapeutic windows differ markedly between individuals and between mechanistic pathways (e.g., oxidative stress vs. inflammation vs. ER stress).

Ultimately, precision medicine in TBI aims to transition from population-based averages toward data-informed individual therapeutics, maximizing efficacy while minimizing off-target effects (Vilkaite et al., 2024). Yet, successful implementation will require standardized biomarker pipelines, cross-center data harmonization, and integration of multi-modal stress-response readouts to fully capture the mechanistic heterogeneity of TBI.

### 6.3 Artificial intelligence and imaging-based prognostics

The integration of artificial intelligence (AI) and advanced neuroimaging offers unprecedented potential for improving prognosis, disease monitoring, and treatment optimization after TBI (Uparela-Reyes et al., 2024). AI algorithms can synthesize multimodal data—including MRI, CT, electrophysiology, and molecular biomarkers—to extract latent patterns that predict functional recovery trajectories (Mohsen et al., 2022). In TBI, where injury mechanisms vary widely across patients, AI tools are particularly valuable for capturing subtle microstructural and metabolic abnormalities that traditional analyses often overlook.

Deep-learning frameworks trained on large-scale imaging datasets can detect subtle microstructural abnormalities, white-matter disconnection, and diffuse axonal injury, which are often invisible to conventional analyses (Onciul et al., 2025). These signatures correlate with stress-response mechanisms described earlier—including mitochondrial energy failure, axonal cytoskeletal breakdown, and inflammation-driven white-matter degeneration—linking radiological features to the underlying molecular pathology of TBI.

When combined with omics-based biomarkers, AI-driven imaging analytics enable spatially resolved mapping of injury heterogeneity, linking molecular signatures to structural and functional alterations (Ibrahim et al., 2025). For example, imaging-detected axonal fragility may correspond to elevated NfL levels, while regions showing reduced functional connectivity may align with transcriptomic signatures of ER stress, inflammasome activation, or impaired mitochondrial metabolism. This multimodal integration is essential for identifying TBI subtypes defined by distinct stress-pathway dominance.

Furthermore, predictive AI models can support clinical decision-making, identifying high-risk patients and tailoring rehabilitation strategies accordingly (Irannejad et al., 2025). Yet, many such models have been trained on datasets from mixed neurological conditions or limited single-center cohorts, raising concerns about generalizability and performance in diverse TBI populations. Differences in scanner protocols, demographic variables, and injury biomechanics can significantly affect model outputs, limiting immediate clinical translation.

Future developments in explainable AI, federated learning, and real-time predictive imaging will enhance model transparency, data security, and clinical applicability across institutions. Explainable AI will be critical for linking model predictions to interpretable features such as MAM-associated metabolic decline, mtROS-sensitive white-matter injury, or Ca^2+^-related functional network disruptions. Federated learning may help address privacy constraints while enabling training on much larger and more heterogeneous TBI datasets.

Collectively, the convergence of AI, multi-omics, and neuroimaging is expected to establish an integrated precision platform, enabling dynamic and individualized prediction of outcomes following brain injury (Alshehri, 2025). Nonetheless, successful deployment will require rigorous validation across diverse cohorts, standardized imaging pipelines, and mechanistic anchoring of AI predictions to biologically meaningful stress pathways.

## 7 Conclusion

### 7.1 Emphasizing stress crosstalk as a novel target for TBI therapy

Recent studies highlights the pathological interdependence of oxidative stress, neuroinflammation, and mitochondrial dysfunction in TBI and neurodegenerative disorders. Rather than acting in isolation, these processes form complex, mutually reinforcing networks that amplify neuronal damage and impair recovery. Stress crosstalk mechanisms—such as ROS-ER feedback loops, UPR-mitochondrial axis, and inflammation-driven metabolic reprogramming—represent promising therapeutic targets. Interventions designed to simultaneously modulate multiple stress pathways, such as combined antioxidants and ER stress inhibitors, or nanoparticle-delivered siRNA targeting master regulators like ATF4 or Nrf2, may offer synergistic benefits. Recognizing and targeting these stress integration points could shift the therapeutic paradigm from symptom management to root cause intervention in TBI.

### 7.2 Interdisciplinary collaboration and clinical translation

To significantly advance the translation of molecular insights into clinically effective therapies, there exists an urgent need to maintain long-term interdisciplinary interaction among neuroscience, systems biology, biomedical engineering, and clinical medicine. Intersectoral alliances, which consist of the academia, industry players, and regulatory bodies, are critical in the advancement, authorization, and practice of up-coming technology in diagnostics and therapeutics. As an example, application of the AI-based neuroimaging biomarkers requires a frictionless collaboration between computational scientists and the neuroradiologists whereas clinical implementation of gene therapies requires a synergized skill set covering nanotechnology, pharmacology, and clinical study design. Also, it is crucial to incorporate patient-centered clinical research programs, real-world evidence, and efficient regulatory processes in order to speed up translational pipelines. International networks that deal with data-sharing, qualification of biomarkers, and standardization of protocols will be able to match the complexity and the heterogeneity that are inherent to neurodegenerative diseases and to TBI. In the future, a systems-level approach will be useful by incorporating design elements from mechanistic explorations with engineering innovations and practice-oriented interventional tactics in treating patients across the globe.

## References

[ref1] Alam A. Thelin E. P. Tajsic T. Khan D. Z. Khellaf A. Patani R. . (2020). Cellular infiltration in traumatic brain injury. J. Neuroinflammation 17:328. doi: 10.1186/s12974-020-02005-x, 33143727 PMC7640704

[ref2] Al-Khateeb Z. F. Boumenar H. Adebimpe J. Shekerzade S. Henson S. M. Tremoleda J. L. . (2024). The cellular senescence response and neuroinflammation in juvenile mice following controlled cortical impact and repetitive mild traumatic brain injury. Exp. Neurol. 374:114714. doi: 10.1016/j.expneurol.2024.114714, 38325653

[ref3] Alshehri Z. S. (2025). Integrating artificial intelligence with small molecule therapeutics and precision medicine for neurochemical understanding of Alzheimer's diseases. Neuroscience 586, 44–57. doi: 10.1016/j.neuroscience.2025.08.055, 40975511

[ref4] Amato L. G. Lassi M. Vergani A. A. . (2025). Digital twins and non-invasive recordings enable early diagnosis of Alzheimer’s disease. Alzheimer's Res Ther 17:125. doi: 10.1186/s13195-025-01765-z, 40450374 PMC12125947

[ref5] Anash M. Maparu K. Singh S. (2025). Unraveling cell death mechanisms in traumatic brain injury: dynamic roles of ferroptosis and necroptosis. Mol. Biol. Rep. 52:381. doi: 10.1007/s11033-025-10489-0, 40208458

[ref6] Bahuguna D. Amulya E. Arya S. Loharkar S. Vambhurkar G. Bhattacharjee S. . (2025). Unlocking therapeutic potential in traumatic brain injury: exploring microenvironmental targets, signaling pathways and translational hurdles. Inflammopharmacology 33, 5113–5144. doi: 10.1007/s10787-025-01923-7, 40889011

[ref7] Bains M. Cebak J. E. Gilmer L. K. Barnes C. C. Thompson S. N. Geddes J. W. . (2013). Pharmacological analysis of the cortical neuronal cytoskeletal protective efficacy of the calpain inhibitor SNJ-1945 in a mouse traumatic brain injury model. J. Neurochem. 125, 125–132. doi: 10.1111/jnc.12118, 23216523

[ref8] Bano N. Khan S. Ahamad S. Dar N. J. Alanazi H. H. Nazir A. . (2025). Microglial NOX2 as a therapeutic target in traumatic brain injury: mechanisms, consequences, and potential for neuroprotection. Ageing Res. Rev. 108:102735. doi: 10.1016/j.arr.2025.102735, 40122395

[ref9] Baral H. Kaundal R. K. (2025). Novel insights into neuroinflammatory mechanisms in traumatic brain injury: focus on pattern recognition receptors as therapeutic targets. Cytokine Growth Factor Rev. 83, 18–34. doi: 10.1016/j.cytogfr.2025.03.001, 40169306

[ref10] Baudry M. Luo Y. L. Bi X. (2023). Calpain-2 inhibitors as therapy for traumatic brain injury. Neurotherapeutics 20, 1592–1602. doi: 10.1007/s13311-023-01407-y, 37474874 PMC10684478

[ref11] Bellotti C. Samudyata S. Thams S. Sellgren C. M. Rostami E. (2024). Organoids and chimeras: the hopeful fusion transforming traumatic brain injury research. Acta Neuropathol. Commun. 12:141. doi: 10.1186/s40478-024-01845-5, 39215375 PMC11363608

[ref12] Bellou E. Kim W. Leonenko G. Tao F. Simmonds E. Wu Y. . (2025). Benchmarking Alzheimer's disease prediction: personalised risk assessment using polygenic risk scores across various methodologies and genome-wide studies. Alzheimer's Res Ther 17:6. doi: 10.1186/s13195-024-01664-9, 39762974 PMC11702271

[ref13] Bergold P. J. Furhang R. Lawless S. (2023). Treating traumatic brain injury with minocycline. Neurotherapeutics 20, 1546–1564. doi: 10.1007/s13311-023-01426-9, 37721647 PMC10684850

[ref14] Blennow K. Zetterberg H. (2018). Biomarkers for Alzheimer's disease: current status and prospects for the future. J. Intern. Med. 284, 643–663. doi: 10.1111/joim.12816, 30051512

[ref15] Bras J. P. Bravo J. Freitas J. Barbosa M. A. Santos S. G. Summavielle T. . (2020). TNF-alpha-induced microglia activation requires miR-342: impact on NF-kB signaling and neurotoxicity. Cell Death Dis. 11:415. doi: 10.1038/s41419-020-2626-6, 32488063 PMC7265562

[ref16] Caceres E. Olivella J. C. Di Napoli M. Raihane A. S. Divani A. A. (2024). Immune response in traumatic brain injury. Curr. Neurol. Neurosci. Rep. 24, 593–609. doi: 10.1007/s11910-024-01382-7, 39467990 PMC11538248

[ref17] Cai Y. Sui L. Wang J. Qian W. Peng Y. Gong L. . (2024). Post-marketing surveillance framework of cell and gene therapy products in the European Union, the United States, Japan, South Korea and China: a comparative study. BMC Med. 22:421. doi: 10.1186/s12916-024-03637-z, 39334246 PMC11438358

[ref18] Canal-Garcia A. Vereb D. Mijalkov M. . (2024). Dynamic multilayer functional connectivity detects preclinical and clinical Alzheimer’s disease. Cereb. Cortex 34:542. doi: 10.1093/cercor/bhad542, 38212285 PMC10839846

[ref19] Chakraborty R. Tabassum H. Parvez S. (2023). NLRP3 inflammasome in traumatic brain injury: its implication in the disease pathophysiology and potential as a therapeutic target. Life Sci. 314:121352. doi: 10.1016/j.lfs.2022.121352, 36592789

[ref20] Chaplin A. Gao H. Asase C. Rengasamy P. Park B. Skander D. . (2020). Systemically-delivered biodegradable PLGA alters gut microbiota and induces transcriptomic reprogramming in the liver in an obesity mouse model. Sci. Rep. 10:13786. doi: 10.1038/s41598-020-69745-x, 32796856 PMC7429827

[ref21] Chen Y. Huang J. Li Y. Chen Y. Gong Z. Xu M. . (2024). Bongkrekic acid alleviates airway inflammation via breaking the mPTP/mtDAMPs/RAGE feedback loop in a steroid-insensitive asthma model. Biomed. Pharmacother. 177:117111. doi: 10.1016/j.biopha.2024.117111, 39013220

[ref22] Chen X. Shi C. He M. Xiong S. Xia X. (2023). Endoplasmic reticulum stress: molecular mechanism and therapeutic targets. Signal Transduct. Target. Ther. 8:352. doi: 10.1038/s41392-023-01570-w, 37709773 PMC10502142

[ref23] Chen P. Zhang S. Zhao K. Kang X. Rittman T. Liu Y. (2024). Robustly uncovering the heterogeneity of neurodegenerative disease by using data-driven subtyping in neuroimaging: a review. Brain Res. 1823:148675. doi: 10.1016/j.brainres.2023.148675, 37979603

[ref24] Cheng G. Liu Y. Ma R. Cheng G. Guan Y. Chen X. . (2022). Anti-parkinsonian therapy: strategies for crossing the blood-brain barrier and nano-biological effects of nanomaterials. Nanomicro Lett. 14:105. doi: 10.1007/s40820-022-00847-z, 35426525 PMC9012800

[ref25] Chuckowree J. A. Zhu Z. Brizuela M. Lee K. M. Blizzard C. A. Dickson T. C. (2018). The microtubule-modulating drug epothilone d alters dendritic spine morphology in a mouse model of mild traumatic brain injury. Front. Cell. Neurosci. 12:223. doi: 10.3389/fncel.2018.00223, 30104961 PMC6077201

[ref26] Clark R. S. B. Empey P. E. Kochanek P. M. Bell M. J. (2023). N-acetylcysteine and probenecid adjuvant therapy for traumatic brain injury. Neurotherapeutics 20, 1529–1537. doi: 10.1007/s13311-023-01422-z, 37596428 PMC10684451

[ref27] Cruceriu D. Baldasici O. Balacescu O. Berindan-Neagoe I. (2020). The dual role of tumor necrosis factor-alpha (TNF-alpha) in breast cancer: molecular insights and therapeutic approaches. Cell. Oncol. (Dordr) 43, 1–18. doi: 10.1007/s13402-019-00489-1, 31900901 PMC12990688

[ref28] Cruz Navarro J. Ponce Mejia L. L. Robertson C. (2022). A precision medicine agenda in traumatic brain injury. Front. Pharmacol. 13:713100. doi: 10.3389/fphar.2022.713100, 35370671 PMC8966615

[ref29] Daniore P. Nittas V. Haag C. Bernard J. Gonzenbach R. von Wyl V. (2024). From wearable sensor data to digital biomarker development: ten lessons learned and a framework proposal. NPJ Digit Med. 7:161. doi: 10.1038/s41746-024-01151-3, 38890529 PMC11189504

[ref30] Davis C. K. Bathula S. Hsu M. Morris-Blanco K. C. Chokkalla A. K. Jeong S. . (2022). An antioxidant and anti-ER stress combo therapy decreases inflammation, secondary brain damage and promotes neurological recovery following traumatic brain injury in mice. J. Neurosci. 42, 6810–6821. doi: 10.1523/JNEUROSCI.0212-22.2022, 35882557 PMC9436019

[ref31] Davis C. K. Vemuganti R. (2021). DNA damage and repair following traumatic brain injury. Neurobiol. Dis. 147:105143. doi: 10.1016/j.nbd.2020.105143, 33127471

[ref32] de Macedo Filho L. Figueredo L. F. Villegas-Gomez G. A. Arthur M. Pedraza-Ciro M. C. Martins H. . (2024). Pathophysiology-based management of secondary injuries and insults in TBI. Biomedicine 12:520. doi: 10.3390/biomedicines12030520, 38540133 PMC10968249

[ref33] de Rojas I. Moreno-Grau S. Tesi N. Grenier-Boley B. Andrade V. Jansen I. E. . (2021). Common variants in Alzheimer's disease and risk stratification by polygenic risk scores. Nat. Commun. 12:3417. doi: 10.1038/s41467-021-22491-8, 34099642 PMC8184987

[ref34] Delint-Ramirez I. Madabhushi R. (2025). DNA damage and its links to neuronal aging and degeneration. Neuron 113, 7–28. doi: 10.1016/j.neuron.2024.12.001, 39788088 PMC11832075

[ref35] Delpech J. C. Valdearcos M. Nadjar A. (2024). Stress and microglia: a double-edged relationship. Adv Neurobiol 37, 333–342. doi: 10.1007/978-3-031-55529-9_18 39207700

[ref36] Dhir N. Jain A. Sharma A. R. Prakash A. Radotra B. D. Medhi B. (2023). PERK inhibitor, GSK2606414, ameliorates neuropathological damage, memory and motor functional impairments in cerebral ischemia via PERK/p-eIF2a/ATF4/CHOP signaling. Metab. Brain Dis. 38, 1177–1192. doi: 10.1007/s11011-023-01183-w, 36847967

[ref37] Ding Q. Chaplin J. Morris M. J. Hilliard M. A. Wolvetang E. Ng D. C. H. . (2021). TDP-43 mutation affects stress granule dynamics in differentiated NSC-34 motoneuron-like cells. Front. Cell Dev. Biol. 9:611601. doi: 10.3389/fcell.2021.611601, 34169068 PMC8217991

[ref38] Dogan E. O. Bouley J. Zhong J. Harkins A. L. Keeler A. M. Bosco D. A. . (2023). Genetic ablation of Sarm1 attenuates expression and mislocalization of phosphorylated TDP-43 after mouse repetitive traumatic brain injury. Acta Neuropathol. Commun. 11:206. doi: 10.1186/s40478-023-01709-4, 38124145 PMC10731794

[ref39] Egan M. F. Kost J. Tariot P. N. Aisen P. S. Cummings J. L. Vellas B. . (2018). Randomized trial of verubecestat for mild-to-moderate Alzheimer's disease. N. Engl. J. Med. 378, 1691–1703. doi: 10.1056/NEJMoa1706441, 29719179 PMC6776074

[ref40] Endlicher R. Drahota Z. Stefkova K. Cervinkova Z. Kucera O. (2023). The mitochondrial permeability transition pore-current knowledge of its structure, function, and regulation, and optimized methods for evaluating its functional state. Cells 12:1273. doi: 10.3390/cells12091273, 37174672 PMC10177258

[ref41] Erickson C. M. Wexler A. Largent E. A. (2025). Digital biomarkers for neurodegenerative disease. JAMA Neurol. 82, 5–6. doi: 10.1001/jamaneurol.2024.3533, 39432291 PMC11729353

[ref42] Eteleeb A. M. Novotny B. C. Tarraga C. S. Sohn C. Dhungel E. Brase L. . (2024). Brain high-throughput multi-omics data reveal molecular heterogeneity in Alzheimer's disease. PLoS Biol. 22:e3002607. doi: 10.1371/journal.pbio.3002607, 38687811 PMC11086901

[ref43] Fakhri S. Piri S. Moradi S. Z. Khan H. (2022). Phytochemicals targeting oxidative stress, interconnected neuroinflammatory, and neuroapoptotic pathways following radiation. Curr. Neuropharmacol. 20, 836–856. doi: 10.2174/1570159X19666210809103346, 34370636 PMC9881105

[ref44] Fedoruk R. P. Lee C. H. Banoei M. M. Winston B. W. (2023). Metabolomics in severe traumatic brain injury: a scoping review. BMC Neurosci. 24:54. doi: 10.1186/s12868-023-00824-1, 37845610 PMC10577974

[ref45] Feng Y. Ju Y. Wu Q. Sun G. Yan Z. (2023). TAK-242, a toll-like receptor 4 antagonist, against brain injury by alleviates autophagy and inflammation in rats. Open Life Sci. 18:20220662. doi: 10.1515/biol-2022-0662, 37528888 PMC10389675

[ref46] Feng Y. Ju Y. Yan Z. Ji M. Li J. Wu Q. . (2022). Resveratrol attenuates autophagy and inflammation after traumatic brain injury by activation of PI3K/Akt/mTOR pathway in rats. Folia Neuropathol. 60, 153–164. doi: 10.5114/fn.2022.118184, 35950468

[ref47] Fernandez-Gajardo R. Matamala J. M. Carrasco R. Gutierrez R. Melo R. Rodrigo R. (2014). Novel therapeutic strategies for traumatic brain injury: acute antioxidant reinforcement. CNS Drugs 28, 229–248. doi: 10.1007/s40263-013-0138-y, 24532027

[ref48] Fesharaki-Zadeh A. (2022). Oxidative stress in traumatic brain injury. Int. J. Mol. Sci. 23, 1201–1211. doi: 10.3390/ijms232113000, 36361792 PMC9657447

[ref49] Fields M. Marcuzzi A. Gonelli A. Celeghini C. Maximova N. Rimondi E. (2023). Mitochondria-targeted antioxidants, an innovative class of antioxidant compounds for neurodegenerative diseases: perspectives and limitations. Int. J. Mol. Sci. 24:2739. doi: 10.3390/ijms24043739, 36835150 PMC9960436

[ref50] Filippone A. Esposito E. Mannino D. Lyssenko N. Pratico D. (2022). The contribution of altered neuronal autophagy to neurodegeneration. Pharmacol. Ther. 238:108178. doi: 10.1016/j.pharmthera.2022.108178, 35351465 PMC9510148

[ref51] Foley K. E. Wilcock D. M. (2024). Three major effects of APOEε4 on aβ immunotherapy induced ARIA. Front. Aging Neurosci. 16:1412006. doi: 10.3389/fnagi.2024.1412006, 38756535 PMC11096466

[ref52] Fu H. Cheng J. Hu L. Heng B. C. Zhang X. Deng X. . (2025). Mitochondria-targeting materials and therapies for regenerative engineering. Biomaterials 316:123023. doi: 10.1016/j.biomaterials.2024.123023, 39708774

[ref53] Fu X. Zhang Y. Chen G. Mao G. Tang J. Xu J. . (2025). Responsive nanoparticles synergize with curcumin to break the "reactive oxygen species-Neuroinflammation" vicious cycle, enhancing traumatic brain injury outcomes. J Nanobiotechnology. 23:172. doi: 10.1186/s12951-025-03251-y, 40045354 PMC11881390

[ref54] Furukawa Y. Megata M. Shintani A. Sue K. Morohoshi T. Akutsu M. . (2025). Cu/Zn-superoxide dismutase naturally fused with a β-propeller lactonase in *Deinococcus radiodurans*. J. Biol. Chem. 301:110499. doi: 10.1016/j.jbc.2025.110499, 40684944 PMC12362111

[ref55] Golovachev N. Siebold L. Sutton R. L. Ghavim S. Harris N. G. Bartnik-Olson B. (2024). Metabolic-driven analytics of traumatic brain injury and neuroprotection by ethyl pyruvate. J. Neuroinflammation 21:294. doi: 10.1186/s12974-024-03280-8, 39538295 PMC11562096

[ref56] Gottschalk B. Koshenov Z. Bachkoenig O. A. Rost R. Malli R. Graier W. F. (2022). MFN2 mediates ER-mitochondrial coupling during ER stress through specialized stable contact sites. Front. Cell Dev. Biol. 10:918691. doi: 10.3389/fcell.2022.918691, 36158213 PMC9493370

[ref57] Gouda A. R. El-Bassiouny N. A. Salahuddin A. Hamouda E. H. Kassem A. B. (2025). Repurposing of high-dose N-acetylcysteine as anti-inflammatory, antioxidant and neuroprotective agent in moderate to severe traumatic brain injury patients: a randomized controlled trial. Inflammopharmacology 33, 3307–3316. doi: 10.1007/s10787-025-01706-0, 40205270 PMC12213923

[ref58] Grovola M. R. Paleologos N. Brown D. P. Tran N. Wofford K. L. Harris J. P. . (2021). Diverse changes in microglia morphology and axonal pathology during the course of 1 year after mild traumatic brain injury in pigs. Brain Pathol. 31:e12953. doi: 10.1111/bpa.12953, 33960556 PMC8412066

[ref59] Grujicic J. Allen A. R. (2024). MnSOD mimetics in therapy: exploring their role in combating oxidative stress-related diseases. Antioxidants (Basel). 13:1444. doi: 10.3390/antiox13121444, 39765773 PMC11672822

[ref60] Gu L. Zhang L. Li C. Jiang L. Zhou J. Xie Y. . (2025). Global, regional, and national burden of traumatic brain injury, 1990-2021: a systematic analysis for the global burden of disease study 2021. J. Neurotrauma 42, 1805–1815. doi: 10.1089/neu.2025.0039, 40622274

[ref61] Guo J. Li Z. Yao Y. Fang L. Yu M. Wang Z. (2024). Curcumin in the treatment of inflammation and oxidative stress responses in traumatic brain injury: a systematic review and meta-analysis. Front. Neurol. 15:1380353. doi: 10.3389/fneur.2024.1380353, 38798711 PMC11116723

[ref62] Ha D. P. Shin W. J. Liu Z. Doche M. E. Lau R. Leli N. M. . (2024). Targeting stress induction of GRP78 by cardiac glycoside oleandrin dually suppresses cancer and COVID-19. Cell Biosci. 14:115. doi: 10.1186/s13578-024-01297-3, 39238058 PMC11378597

[ref63] Hafycz J. M. Strus E. Naidoo N. N. (2023). Early and late chaperone intervention therapy boosts XBP1s and ADAM10, restores proteostasis, and rescues learning in Alzheimer's disease mice. bioRxiv 2023:2023.2005.2023.541973. doi: 10.1101/2023.05.23.541973, 37292838 PMC10245863

[ref64] Hahm J. Y. Park J. Jang E. S. Chi S. W. (2022). 8-Oxoguanine: from oxidative damage to epigenetic and epitranscriptional modification. Exp. Mol. Med. 54, 1626–1642. doi: 10.1038/s12276-022-00822-z, 36266447 PMC9636213

[ref65] Haidar M. A. Shakkour Z. Barsa C. Tabet M. Mekhjian S. Darwish H. . (2022). Mitoquinone helps combat the neurological, cognitive, and molecular consequences of open head traumatic brain injury at chronic time point. Biomedicine 10:250. doi: 10.3390/biomedicines10020250, 35203460 PMC8869514

[ref66] Hampel H. Cummings J. Blennow K. Gao P. Jack C. R. Jr. Vergallo A. (2021). Developing the ATX(N) classification for use across the Alzheimer disease continuum. Nat. Rev. Neurol. 17, 580–589. doi: 10.1038/s41582-021-00520-w, 34239130

[ref67] Hampel H. Gao P. Cummings J. Toschi N. Thompson P. M. Hu Y. . (2023). The foundation and architecture of precision medicine in neurology and psychiatry. Trends Neurosci. 46, 176–198. doi: 10.1016/j.tins.2022.12.004, 36642626 PMC10720395

[ref68] Hao L. Yang Y. Xu X. Guo X. Zhan Q. (2022). Modulatory effects of mesenchymal stem cells on microglia in ischemic stroke. Front. Neurol. 13:1073958. doi: 10.3389/fneur.2022.1073958, 36742051 PMC9889551

[ref69] Harned T. C. Stan R. V. Cao Z. Chakrabarti R. Higgs H. N. Chang C. C. Y. . (2023). Acute ACAT1/SOAT1 blockade increases MAM cholesterol and strengthens ER-mitochondria connectivity. Int. J. Mol. Sci. 24:5525. doi: 10.3390/ijms24065525, 36982602 PMC10059652

[ref70] He X. Liu X. Zuo F. Shi H. Jing J. (2023). Artificial intelligence-based multi-omics analysis fuels cancer precision medicine. Semin. Cancer Biol. 88, 187–200. doi: 10.1016/j.semcancer.2022.12.009, 36596352

[ref71] He Y. Wang Y. Wang L. Jiang W. Wilhelm S. (2024). Understanding nanoparticle-liver interactions in nanomedicine. Expert Opin. Drug Deliv. 21, 829–843. doi: 10.1080/17425247.2024.2375400, 38946471 PMC11281865

[ref72] Helmrich I. Czeiter E. Amrein K. Büki A. Lingsma H. F. Menon D. K. . (2022). Incremental prognostic value of acute serum biomarkers for functional outcome after traumatic brain injury (CENTER-TBI): an observational cohort study. Lancet Neurol. 21, 792–802. doi: 10.1016/S1474-4422(22)00218-6, 35963262

[ref73] Hernandez D. Morgan Schlicht S. Elli Clarke J. Daniszewski M. Karch C. M. Adams S. . (2024). Generation of a gene-corrected human isogenic iPSC line from an Alzheimer's disease iPSC line carrying the PSEN1 H163R mutation. Stem Cell Res. 79:103495. doi: 10.1016/j.scr.2024.103495, 39079290 PMC11608089

[ref74] Hinderer C. Katz N. Buza E. L. Dyer C. Goode T. Bell P. . (2018). Severe toxicity in nonhuman primates and piglets following high-dose intravenous administration of an adeno-associated virus vector expressing human SMN. Hum. Gene Ther. 29, 285–298. doi: 10.1089/hum.2018.015, 29378426 PMC5865262

[ref75] Hood K. N. Zhao J. Redell J. B. Hylin M. J. Harris B. Perez A. . (2018). Endoplasmic reticulum stress contributes to the loss of newborn hippocampal neurons after traumatic brain injury. J. Neurosci. 38, 2372–2384. doi: 10.1523/JNEUROSCI.1756-17.2018, 29386258 PMC5830522

[ref76] Houle S. Kokiko-Cochran O. N. (2021). A levee to the flood: pre-injury neuroinflammation and immune stress influence traumatic brain injury outcome. Front. Aging Neurosci. 13:788055. doi: 10.3389/fnagi.2021.788055, 35095471 PMC8790486

[ref77] Huang Z. Merrihew G. E. Larson E. B. Park J. Plubell D. Fox E. J. . (2023). Brain proteomic analysis implicates actin filament processes and injury response in resilience to Alzheimer's disease. Nat. Commun. 14:2747. doi: 10.1038/s41467-023-38376-x, 37173305 PMC10182086

[ref78] Huang Y. Wu L. Zhao Y. Guo J. Li R. Ma S. . (2024). Schwann cell promotes macrophage recruitment through IL-17B/IL-17RB pathway in injured peripheral nerves. Cell Rep. 43:113753. doi: 10.1016/j.celrep.2024.113753, 38341853

[ref79] Huo J. Molkentin J. D. (2024). MCU genetically altered mice suggest how mitochondrial Ca^2+^ regulates metabolism. Trends Endocrinol. Metab. 35, 918–928. doi: 10.1016/j.tem.2024.04.005, 38688781 PMC11490413

[ref80] Hurley E. M. Mozolewski P. Dobrowolski R. Hsieh J. (2023). Familial Alzheimer's disease-associated PSEN1 mutations affect neurodevelopment through increased notch signaling. Stem Cell Reports. 18, 1516–1533. doi: 10.1016/j.stemcr.2023.05.018, 37352850 PMC10362499

[ref81] Ibrahim M. Khalil Y. A. Amirrajab S. Sun C. Breeuwer M. Pluim J. . (2025). Generative AI for synthetic data across multiple medical modalities: a systematic review of recent developments and challenges. Comput. Biol. Med. 189:109834. doi: 10.1016/j.compbiomed.2025.109834, 40023073

[ref82] Ionescu C. Ghidersa M. Ciobica A. Mavroudis I. Kazis D. Petridis F. E. . (2025). Potential correlation between molecular biomarkers and oxidative stress in traumatic brain injury. Int. J. Mol. Sci. 26:3858. doi: 10.3390/ijms26083858, 40332547 PMC12027598

[ref83] Irannejad K. Mafi M. Krishnan S. Budoff M. J. (2025). Artificial intelligence in coronary CT angiography: transforming the diagnosis and risk stratification of atherosclerosis. Int. J. Cardiovasc. Imaging 41, 1643–1656. doi: 10.1007/s10554-025-03440-8, 40576859

[ref84] Jamjoom A. A. B. Rhodes J. Andrews P. J. D. Grant S. G. N. (2021). The synapse in traumatic brain injury. Brain 144, 18–31. doi: 10.1093/brain/awaa321, 33186462 PMC7880663

[ref85] Jeon Y. M. Kwon Y. Lee S. Kim H. J. (2023). Potential roles of the endoplasmic reticulum stress pathway in amyotrophic lateral sclerosis. Front. Aging Neurosci. 15:1047897. doi: 10.3389/fnagi.2023.1047897, 36875699 PMC9974850

[ref86] Kalra S. Malik R. Singh G. Bhatia S. al-Harrasi A. Mohan S. . (2022). Pathogenesis and management of traumatic brain injury (TBI): role of neuroinflammation and anti-inflammatory drugs. Inflammopharmacology 30, 1153–1166. doi: 10.1007/s10787-022-01017-8, 35802283 PMC9293826

[ref87] Kang K. Chen S. H. Wang D. P. Chen F. (2024). Inhibition of endoplasmic reticulum stress improves chronic ischemic hippocampal damage associated with suppression of IRE1alpha/TRAF2/ASK1/JNK-dependent apoptosis. Inflammation 47, 1479–1490. doi: 10.1007/s10753-024-01989-5, 38401021 PMC11343861

[ref88] Karvandi M. S. Sheikhzadeh Hesari F. Aref A. R. Mahdavi M. (2023). The neuroprotective effects of targeting key factors of neuronal cell death in neurodegenerative diseases: the role of ER stress, oxidative stress, and neuroinflammation. Front. Cell. Neurosci. 17:1105247. doi: 10.3389/fncel.2023.1105247, 36950516 PMC10025411

[ref89] Khalaf K. Tornese P. Cocco A. Albanese A. (2022). Tauroursodeoxycholic acid: a potential therapeutic tool in neurodegenerative diseases. Transl Neurodegener. 11:33. doi: 10.1186/s40035-022-00307-z, 35659112 PMC9166453

[ref90] Khalil M. Teunissen C. E. Lehmann S. Otto M. Piehl F. Ziemssen T. . (2024). Neurofilaments as biomarkers in neurological disorders-towards clinical application. Nat. Rev. Neurol. 20, 269–287. doi: 10.1038/s41582-024-00955-x, 38609644

[ref91] Kim J. Lee H. Lee J. Rhee S. Y. Shin J. I. Lee S. W. . (2023). Quantification of identifying cognitive impairment using olfactory-stimulated functional near-infrared spectroscopy with machine learning: a post hoc analysis of a diagnostic trial and validation of an external additional trial. Alzheimer's Res Ther 15:127. doi: 10.1186/s13195-023-01268-9, 37481573 PMC10362671

[ref92] Kimura N. Sasaki K. Masuda T. . (2025). Machine learning models for dementia screening to classify brain amyloid positivity on positron emission tomography using blood markers and demographic characteristics: a retrospective observational study. Alzheimer's Res Ther 17:25. doi: 10.1186/s13195-024-01650-1, 39838434 PMC11748352

[ref93] Komori S. Cross D. J. Mills M. Ouchi Y. Nishizawa S. Okada H. . (2022). Deep-learning prediction of amyloid deposition from early-phase amyloid positron emission tomography imaging. Ann. Nucl. Med. 36, 913–921. doi: 10.1007/s12149-022-01775-z, 35913591

[ref94] Kress J. K. C. Jessen C. Hufnagel A. Schmitz W. da Xavier Silva T. N. dos Ferreira Santos A. . (2023). The integrated stress response effector ATF4 is an obligatory metabolic activator of NRF2. Cell Rep. 42:112724. doi: 10.1016/j.celrep.2023.112724, 37410595

[ref95] Kumari N. Bagri K. Kumari S. Deshmukh R. (2025). Traumatic Brian injury (TBI) unraveled: molecular disruptions and therapeutic avenues. Inflammopharmacology 33, 4323–4334. doi: 10.1007/s10787-025-01870-3, 40715927

[ref96] Lange S. Inal J. M. (2023). Animal models of human disease. Int. J. Mol. Sci. 24:15821. doi: 10.3390/ijms242115821, 37958801 PMC10650829

[ref97] Larranaga-SanMiguel A. Bengoa-Vergniory N. Flores-Romero H. (2025). Crosstalk between mitochondria-ER contact sites and the apoptotic machinery as a novel health meter. Trends Cell Biol. 35, 33–45. doi: 10.1016/j.tcb.2024.08.007, 39379268

[ref98] Lee Y. Miller M. R. Fernandez M. A. Berg E. L. Prada A. M. Ouyang Q. . (2022). Early lysosome defects precede neurodegeneration with amyloid-β and tau aggregation in NHE6-null rat brain. Brain 145, 3187–3202. doi: 10.1093/brain/awab467, 34928329 PMC10147331

[ref99] Lee A. V. Nestler K. A. Chiappinelli K. B. (2024). Therapeutic targeting of DNA methylation alterations in cancer. Pharmacol. Ther. 258:108640. doi: 10.1016/j.pharmthera.2024.108640, 38570075

[ref100] Lee C. Yoon S. Moon J. O. (2023). Kaempferol suppresses carbon tetrachloride-induced liver damage in rats via the MAPKs/NF-κB and AMPK/Nrf2 signaling pathways. Int. J. Mol. Sci. 24:6900. doi: 10.3390/ijms24086900, 37108064 PMC10138912

[ref101] Li Q. Hu W. Huang Q. Yang J. Li B. Ma K. . (2023). MiR146a-loaded engineered exosomes released from silk fibroin patch promote diabetic wound healing by targeting IRAK1. Signal Transduct. Target. Ther. 8:62. doi: 10.1038/s41392-022-01263-w, 36775818 PMC9922687

[ref102] Li W. Li Y. Zhao J. Liao J. Wen W. Chen Y. . (2024). Release of damaged mitochondrial DNA: a novel factor in stimulating inflammatory response. Pathol. Res. Pract. 258:155330. doi: 10.1016/j.prp.2024.155330, 38733868

[ref103] Li Y. Niu D. Qi K. Liang D. Long X. (2025). An imaging and genetic-based deep learning network for Alzheimer's disease diagnosis. Front. Aging Neurosci. 17:1532470. doi: 10.3389/fnagi.2025.1532470, 40191788 PMC11968703

[ref104] Li Y. Xia X. Wang Y. Zheng J. C. (2022). Mitochondrial dysfunction in microglia: a novel perspective for pathogenesis of Alzheimer's disease. J. Neuroinflammation 19:248. doi: 10.1186/s12974-022-02613-9, 36203194 PMC9535890

[ref105] Li J. J. Xin N. Yang C. Kim B. G. Tavizon L. A. Hong R. . (2025). Unveiling the intercompartmental signaling axis: mitochondrial to ER stress response (MERSR) and its impact on proteostasis. PLoS Genet. 21:e1011700. doi: 10.1371/journal.pgen.1011700, 40338975 PMC12088515

[ref106] Li Z. Yang J. Li J. Zhao S. Jiang S. Liu W. . (2025). Targeted delivery of BACE1 siRNA for synergistic treatment of Alzheimer's disease. Transl Neurodegener. 14:41. doi: 10.1186/s40035-025-00503-7, 40814010 PMC12351871

[ref107] Li H. Yang X. Song Y. Zhu Q. Liao Z. Liang Y. . (2023). PRRSV infection activates NLRP3 inflammasome through inducing cytosolic mitochondrial DNA stress. Vet. Microbiol. 279:109673. doi: 10.1016/j.vetmic.2023.109673, 36764219

[ref108] Li L. Yu X. Sheng C. Jiang X. Zhang Q. Han Y. . (2022). A review of brain imaging biomarker genomics in Alzheimer's disease: implementation and perspectives. Transl Neurodegener. 11:42. doi: 10.1186/s40035-022-00315-z, 36109823 PMC9476275

[ref109] Lin M. Qi X. (2023). Advances and challenges of stimuli-responsive nucleic acids delivery system in gene therapy. Pharmaceutics. 15:1450. doi: 10.3390/pharmaceutics15051450, 37242692 PMC10220631

[ref110] Lindholm D. Wootz H. Korhonen L. (2006). ER stress and neurodegenerative diseases. Cell Death Differ. 13, 385–392. doi: 10.1038/sj.cdd.4401778, 16397584

[ref111] Liu Y. D. Chang Y. H. Xie X. T. Wang X. Y. Ma H. Y. Liu M. C. . (2025). PET imaging unveils neuroinflammatory mechanisms in psychiatric disorders: from microglial activation to therapeutic innovation. Mol. Neurobiol. 62, 15318–15335. doi: 10.1007/s12035-025-05177-w, 40610825 PMC12559163

[ref112] Liu Y. Flamier A. Bell G. W. Diao A. J. Whitfield T. W. Wang H. C. . (2024). MECP2 directly interacts with RNA polymerase II to modulate transcription in human neurons. Neuron 112, 1943–1958 e1910. doi: 10.1016/j.neuron.2024.04.007 38697112

[ref113] Liu H. He S. Li C. Wang J. Zou Q. Liao Y. . (2022). Tetrandrine alleviates inflammation and neuron apoptosis in experimental traumatic brain injury by regulating the IRE1alpha/JNK/CHOP signal pathway. Brain Behav. 12:e2786. doi: 10.1002/brb3.2786 36377337 PMC9759135

[ref114] Liu J. Xu A. Zhao Z. Fang D. Lv W. Li Y. . (2025). The burden of traumatic brain injury, its causes, and future trend predictions in 204 countries and territories (1990-2021): results from the global burden of disease study 2021. Neuroepidemiology, 1–15. doi: 10.1159/000547563 40784335

[ref115] Long T. Li D. Vale G. Jiang Y. Schmiege P. Yang Z. J. . (2024). Molecular insights into human phosphatidylserine synthase 1 reveal its inhibition promotes LDL uptake. Cell 187, 5665–5678.e18. doi: 10.1016/j.cell.2024.08.004 39208797 PMC11455612

[ref116] Long X. Yao X. Jiang Q. Yang Y. He X. Tian W. . (2020). Astrocyte-derived exosomes enriched with miR-873a-5p inhibit neuroinflammation via microglia phenotype modulation after traumatic brain injury. J. Neuroinflammation 17:89. doi: 10.1186/s12974-020-01761-0, 32192523 PMC7082961

[ref117] Losurdo M. Pedrazzoli M. D'Agostino C. Elia C. A. Massenzio F. Lonati E. . (2020). Intranasal delivery of mesenchymal stem cell-derived extracellular vesicles exerts immunomodulatory and neuroprotective effects in a 3xTg model of Alzheimer's disease. Stem Cells Transl. Med. 9, 1068–1084. doi: 10.1002/sctm.19-0327, 32496649 PMC7445021

[ref118] Lotfy A. AboQuella N. M. Wang H. (2023). Mesenchymal stromal/stem cell (MSC)-derived exosomes in clinical trials. Stem Cell Res Ther 14:66. doi: 10.1186/s13287-023-03287-7, 37024925 PMC10079493

[ref119] Lu X. Gong Y. Hu W. Mao Y. Wang T. Sun Z. . (2022). Ultrastructural and proteomic profiling of mitochondria-associated endoplasmic reticulum membranes reveal aging signatures in striated muscle. Cell Death Dis. 13:296. doi: 10.1038/s41419-022-04746-4, 35368021 PMC8976840

[ref120] Lu Y. Li Z. Zhang S. Zhang T. Liu Y. Zhang L. (2023). Cellular mitophagy: mechanism, roles in diseases and small molecule pharmacological regulation. Theranostics 13, 736–766. doi: 10.7150/thno.79876, 36632220 PMC9830443

[ref121] Luo Q. Song Y. Kang J. Wu Y. Wu F. Li Y. . (2024). Retraction notice to: mtROS-mediated Akt/AMPK/mTOR pathway was involved in copper-induced autophagy and it attenuates copper-induced apoptosis in RAW264.7 mouse monocytes [redox biol. 41 (2021) 101912]. Redox Biol. 73:103232. doi: 10.1016/j.redox.2024.103232 38851954 PMC11229533

[ref122] Ma M. Jiang W. Zhou R. (2024). DAMPs and DAMP-sensing receptors in inflammation and diseases. Immunity 57, 752–771. doi: 10.1016/j.immuni.2024.03.002, 38599169

[ref123] Maas A. I. R. Menon D. K. Manley G. T. Abrams M. Åkerlund C. Andelic N. . (2022). Traumatic brain injury: progress and challenges in prevention, clinical care, and research. Lancet Neurol. 21, 1004–1060. doi: 10.1016/S1474-4422(22)00309-X, 36183712 PMC10427240

[ref124] MacDougall G. Brown L. Y. Kantor B. Chiba-Falek O. (2021). The path to progress preclinical studies of age-related neurodegenerative diseases: a perspective on rodent and hiPSC-derived models. Mol. Ther. 29, 949–972. doi: 10.1016/j.ymthe.2021.01.001, 33429080 PMC7934639

[ref125] Madias M. I. Stessman L. N. Warlof S. J. Kudryashev J. A. Kwon E. J. (2024). Spatial measurement and inhibition of calpain activity in traumatic brain injury with an activity-based nanotheranostic platform. ACS Nano 18, 25565–25576. doi: 10.1021/acsnano.4c06052, 39236689 PMC11411711

[ref126] Manczak M. Calkins M. J. Reddy P. H. (2011). Impaired mitochondrial dynamics and abnormal interaction of amyloid beta with mitochondrial protein Drp1 in neurons from patients with Alzheimer's disease: implications for neuronal damage. Hum. Mol. Genet. 20, 2495–2509. doi: 10.1093/hmg/ddr139, 21459773 PMC3109997

[ref127] Marei H. E. Khan M. U. A. Hasan A. (2023). Potential use of iPSCs for disease modeling, drug screening, and cell-based therapy for Alzheimer's disease. Cell. Mol. Biol. Lett. 28:98. doi: 10.1186/s11658-023-00504-2, 38031028 PMC10687886

[ref128] Martins M. Keir H. R. Chalmers J. D. (2023). Endotypes in bronchiectasis: moving towards precision medicine. A narrative review. Pulmonology 29, 505–517. doi: 10.1016/j.pulmoe.2023.03.004, 37030997

[ref129] Martin-Vega A. Cobb M. H. (2023). Navigating the ERK1/2 MAPK cascade. Biomolecules. 13:1555. doi: 10.3390/biom13101555, 37892237 PMC10605237

[ref130] McDonald B. Z. Tarudji A. W. Zhang H. Ryu S. Eskridge K. M. Kievit F. M. (2024). Traumatic brain injury heterogeneity affects cell death and autophagy. Exp. Brain Res. 242, 1645–1658. doi: 10.1007/s00221-024-06856-1, 38789796 PMC12414495

[ref131] McGettigan S. Nolan Y. Ghosh S. O'Mahony D. (2023). The emerging role of blood biomarkers in diagnosis and treatment of Alzheimer's disease. Eur. Geriatr. Med. 14, 913–917. doi: 10.1007/s41999-023-00847-1, 37648817

[ref132] Meng M. Jiang Y. Wang Y. Huo R. Ma N. Shen X. . (2023). <article-title update="added">β-carotene targets IP3R/GRP75/VDAC1-MCU axis to renovate LPS-induced mitochondrial oxidative damage by regulating STIM1. Free Radic. Biol. Med. 205, 25–46. doi: 10.1016/j.freeradbiomed.2023.05.021, 37270031

[ref133] Merighi A. Lossi L. (2022). Endoplasmic reticulum stress signaling and neuronal cell death. Int. J. Mol. Sci. 23:15186. doi: 10.3390/ijms232315186, 36499512 PMC9740965

[ref134] Mira R. G. Quintanilla R. A. Cerpa W. (2023). Mild traumatic brain injury induces mitochondrial calcium overload and triggers the upregulation of NCLX in the hippocampus. Antioxidants (Basel). 12:403. doi: 10.3390/antiox12020403, 36829963 PMC9952386

[ref135] Modi H. R. Musyaju S. Ratcliffe M. Shear D. A. Scultetus A. H. Pandya J. D. (2024). Mitochondria-targeted antioxidant therapeutics for traumatic brain injury. Antioxidants (Basel). 13:303. doi: 10.3390/antiox13030303, 38539837 PMC10967339

[ref136] Mohammed F. S. Omay S. B. Sheth K. N. Zhou J. (2023). Nanoparticle-based drug delivery for the treatment of traumatic brain injury. Expert Opin. Drug Deliv. 20, 55–73. doi: 10.1080/17425247.2023.2152001, 36420918 PMC9983310

[ref137] Mohan A. A. Talwar P. (2025). MAM kinases: physiological roles, related diseases, and therapeutic perspectives-a systematic review. Cell. Mol. Biol. Lett. 30:35. doi: 10.1186/s11658-025-00714-w, 40148800 PMC11951743

[ref138] Mohsen F. Ali H. El Hajj N. Shah Z. (2022). Artificial intelligence-based methods for fusion of electronic health records and imaging data. Sci. Rep. 12:17981. doi: 10.1038/s41598-022-22514-4, 36289266 PMC9605975

[ref139] Mursaleen L. Chan S. H. Y. Noble B. Somavarapu S. Zariwala M. G. (2023). Curcumin and N-acetylcysteine nanocarriers alone or combined with deferoxamine target the mitochondria and protect against neurotoxicity and oxidative stress in a co-culture model of Parkinson’s disease. Antioxidants 12:130. doi: 10.3390/antiox12010130, 36670992 PMC9855117

[ref140] Narmashiri A. Abbaszadeh M. Ghazizadeh A. (2022). The effects of 1-methyl-4-phenyl-1,2,3,6-tetrahydropyridine (MPTP) on the cognitive and motor functions in rodents: a systematic review and meta-analysis. Neurosci. Biobehav. Rev. 140:104792. doi: 10.1016/j.neubiorev.2022.104792, 35872230

[ref141] Nava Lauson C. B. Tiberti S. Corsetto P. A. Conte F. Tyagi P. Machwirth M. . (2023). Linoleic acid potentiates CD8^+^ T cell metabolic fitness and antitumor immunity. Cell Metab. 35, 633–650 e639. doi: 10.1016/j.cmet.2023.02.013 36898381

[ref142] Nemeth D. P. Liu X. Monet M. C. Niu H. Maxey G. Schrier M. S. . (2024). Localization of brain neuronal IL-1R1 reveals specific neural circuitries responsive to immune signaling. J. Neuroinflammation 21:303. doi: 10.1186/s12974-024-03287-1, 39563437 PMC11575132

[ref143] Neuschmid S. Schallerer C. Ehrlich B. E. McGuone D. (2025). Pathological calcium signaling in traumatic brain injury and Alzheimer's disease: from acute neuronal injury to chronic neurodegeneration. Int. J. Mol. Sci. 26:9245. doi: 10.3390/ijms26189245, 41009808 PMC12471116

[ref144] Ngo W. Ahmed S. Blackadar C. Bussin B. Ji Q. Mladjenovic S. M. . (2022). Why nanoparticles prefer liver macrophage cell uptake in vivo. Adv. Drug Deliv. Rev. 185:114238. doi: 10.1016/j.addr.2022.114238, 35367524

[ref145] Niu F. Dong J. Xu X. Zhang B. Liu B. (2019). Mitochondrial division inhibitor 1 prevents early-stage induction of mitophagy and accelerated cell death in a rat model of moderate controlled cortical impact brain injury. World Neurosurg. 122, e1090–e1101. doi: 10.1016/j.wneu.2018.10.236, 30439527

[ref146] Niu J. Wu Z. Xue H. Zhang Y. Gao Q. Li C. . (2021). Sevoflurane post-conditioning alleviated hypoxic-ischemic brain injury in neonatal rats by inhibiting endoplasmic reticulum stress-mediated autophagy via IRE1 signalings. Neurochem. Int. 150:105198. doi: 10.1016/j.neuint.2021.105198, 34601014

[ref147] O'Brien W. T. Pham L. Symons G. F. Monif M. Shultz S. R. McDonald S. J. (2020). The NLRP3 inflammasome in traumatic brain injury: potential as a biomarker and therapeutic target. J. Neuroinflammation 17:104. doi: 10.1186/s12974-020-01778-5, 32252777 PMC7137518

[ref148] Obukohwo O. M. Oreoluwa O. A. Andrew U. O. Williams U. E. (2024). Microglia-mediated neuroinflammation in traumatic brain injury: a review. Mol. Biol. Rep. 51:1073. doi: 10.1007/s11033-024-09995-4, 39425760

[ref149] Olaghere J. Williams D. A. Farrar J. Büning H. Calhoun C. Ho T. . (2025). Scientific advancements in gene therapies: opportunities for global regulatory convergence. Biomedicine 13:758. doi: 10.3390/biomedicines13030758, 40149734 PMC11940732

[ref150] Onciul R. Tataru C. I. Dumitru A. V. Crivoi C. Serban M. Covache-Busuioc R. A. . (2025). Artificial intelligence and neuroscience: transformative synergies in brain research and clinical applications. J. Clin. Med. 14:550. doi: 10.3390/jcm14020550, 39860555 PMC11766073

[ref151] Oris C. Kahouadji S. Bouvier D. Sapin V. (2024). Blood biomarkers for the management of mild traumatic brain injury in clinical practice. Clin. Chem. 70, 1023–1036. doi: 10.1093/clinchem/hvae049, 38656380

[ref152] Ossenkoppele R. van der Flier W. M. (2023). APOE genotype in the era of disease-modifying treatment with monoclonal antibodies against amyloid-beta. JAMA Neurol. 80, 1269–1271. doi: 10.1001/jamaneurol.2023.4046, 37930662

[ref153] Palmqvist S. Janelidze S. Quiroz Y. T. Zetterberg H. Lopera F. Stomrud E. . (2020). Discriminative accuracy of plasma phospho-tau217 for alzheimer disease vs other neurodegenerative disorders. JAMA 324, 772–781. doi: 10.1001/jama.2020.12134, 32722745 PMC7388060

[ref154] Palomes-Borrajo G. Navarro X. Penas C. (2025). Histone acetylation in central and peripheral nervous system injuries and regeneration: epigenetic dynamics and therapeutic perspectives. Int. J. Mol. Sci. 26:6277. doi: 10.3390/ijms26136277, 40650056 PMC12250607

[ref155] Pan J. Wang Y. Chen Y. Zhang C. Deng H. Lu J. . (2025). Emerging strategies against accelerated blood clearance phenomenon of nanocarrier drug delivery systems. J. Nanobiotechnol. 23:138. doi: 10.1186/s12951-025-03209-0, 40001108 PMC11853785

[ref156] Patel S. Pangarkar A. Mahajan S. Majumdar A. (2023). Therapeutic potential of endoplasmic reticulum stress inhibitors in the treatment of diabetic peripheral neuropathy. Metab. Brain Dis. 38, 1841–1856. doi: 10.1007/s11011-023-01239-x, 37289403

[ref157] Peng D. Huang W. Liu R. Zhong W. (2025). From pixels to prognosis: radiomics and AI in Alzheimer's disease management. Front. Neurol. 16:1536463. doi: 10.3389/fneur.2025.1536463, 39944545 PMC11816362

[ref158] Perneczky R. Froelich L. (2025). Clinically meaningful benefit and real-world evidence in Alzheimer's disease research and care. Alzheimers Dement (N Y) 11:e70090. doi: 10.1002/trc2.70090, 40291121 PMC12022225

[ref159] Pihan P. Ellerby L. M. Hetz C. (2025). ER-mitochondria contact sites: a refuge for mitochondrial mRNAs under ER stress. Trends Cell Biol. 35, 541–543. doi: 10.1016/j.tcb.2025.02.002, 40011091

[ref160] Placeres-Uray F. Gorthy A. S. Torres M. D. Atkins C. M. (2025). Inhibition of microglia priming by NLRP3 reduces the impact of early life stress and mild TBI. J. Neuroinflammation 22:185. doi: 10.1186/s12974-025-03512-5, 40676661 PMC12273472

[ref161] Prolo C. Piacenza L. Radi R. (2024). Peroxynitrite: a multifaceted oxidizing and nitrating metabolite. Curr. Opin. Chem. Biol. 80:102459. doi: 10.1016/j.cbpa.2024.102459, 38723343

[ref162] Qi W. Zhu X. Wang B. Shi Y. Dong C. Shen S. . (2025). Alzheimer's disease digital biomarkers multidimensional landscape and AI model scoping review. NPJ Digit Med. 8:366. doi: 10.1038/s41746-025-01640-z, 40523935 PMC12170881

[ref163] Qian F. Zhong Q. Chen Z. (2024). Role of mitochondrial dysfunction in acute traumatic brain injury: evidence from bioinformatics analysis. Heliyon. 10:e31121. doi: 10.1016/j.heliyon.2024.e31121, 38803920 PMC11128910

[ref164] Qin N. Geng A. Xue R. (2022). Activated or impaired: an overview of DNA repair in neurodegenerative diseases. Aging Dis. 13, 987–1004. doi: 10.14336/AD.2021.1212, 35855336 PMC9286913

[ref165] Rakaee M. Tafavvoghi M. Ricciuti B. Alessi J. V. Cortellini A. Citarella F. . (2025). Deep learning model for predicting immunotherapy response in advanced non-small cell lung cancer. JAMA Oncol. 11, 109–118. doi: 10.1001/jamaoncol.2024.5356, 39724105 PMC11843371

[ref166] Recasens M. Almolda B. Perez-Clausell J. Campbell I. L. Gonzalez B. Castellano B. (2021). Chronic exposure to IL-6 induces a desensitized phenotype of the microglia. J. Neuroinflammation 18:31. doi: 10.1186/s12974-020-02063-1, 33482848 PMC7821504

[ref167] Reicher L. Shilo S. Godneva A. Lutsker G. Zahavi L. Shoer S. . (2025). Deep phenotyping of health-disease continuum in the human phenotype project. Nat. Med. 31, 3191–3203. doi: 10.1038/s41591-025-03790-9, 40665053

[ref168] Rossi S. L. Subramanian P. Bovenkamp D. E. (2023). The future is precision medicine-guided diagnoses, preventions and treatments for neurodegenerative diseases. Front. Aging Neurosci. 15:1128619. doi: 10.3389/fnagi.2023.1128619, 37009453 PMC10065404

[ref169] Ryan A. K. Rich W. Reilly M. A. (2023). Oxidative stress in the brain and retina after traumatic injury. Front. Neurosci. 17:1021152. doi: 10.3389/fnins.2023.1021152, 36816125 PMC9935939

[ref170] Salech F. Ponce D. P. Paula-Lima A. C. SanMartin C. D. Behrens M. I. (2020). Nicotinamide, a poly [ADP-ribose] polymerase 1 (PARP-1) inhibitor, as an adjunctive therapy for the treatment of alzheimer's disease. Front. Aging Neurosci. 12:255. doi: 10.3389/fnagi.2020.00255, 32903806 PMC7438969

[ref171] Salomonsson S. E. Clelland C. D. (2024). Building CRISPR gene therapies for the central nervous system: a review. JAMA Neurol. 81, 283–290. doi: 10.1001/jamaneurol.2023.4983, 38285472 PMC11164426

[ref172] Sapin V. Gaulmin R. Aubin R. Walrand S. Coste A. Abbot M. (2021). Blood biomarkers of mild traumatic brain injury: state of art. Neurochirurgie 67, 249–254. doi: 10.1016/j.neuchi.2021.01.001, 33482234

[ref173] Sassano M. L. van Vliet A. R. Vervoort E. van Eygen S. van den Haute C. Pavie B. . (2023). PERK recruits E-Syt1 at ER-mitochondria contacts for mitochondrial lipid transport and respiration. J. Cell Biol. 222:e202206008. doi: 10.1083/jcb.202206008, 36821088 PMC9998969

[ref174] Satoh R. Utianski R. L. Duffy J. R. Clark H. M. Stephens Y. C. Lee J. . (2025). Distinct 11C-ER176 pet neuroinflammatory profiles and tau colocalization in progressive apraxia of speech with and without Parkinson-plus syndrome. Clin. Nucl. Med. 50, 731–742. doi: 10.1097/RLU.0000000000005962, 40587210 PMC12351688

[ref175] Schallerer C. Neuschmid S. Ehrlich B. E. McGuone D. (2025). Calpain in traumatic brain injury: from cinderella to central player. Cells 14:1253. doi: 10.3390/cells14161253, 40862734 PMC12384584

[ref176] Shahim P. Politis A. van der Merwe A. Moore B. Chou Y. Y. Pham D. L. . (2020). Neurofilament light as a biomarker in traumatic brain injury. Neurology 95, e610–e622. doi: 10.1212/WNL.0000000000009983, 32641538 PMC7455357

[ref177] Shao F. Wang X. Wu H. Wu Q. Zhang J. (2022). Microglia and neuroinflammation: crucial pathological mechanisms in traumatic brain injury-induced neurodegeneration. Front. Aging Neurosci. 14:825086. doi: 10.3389/fnagi.2022.825086, 35401152 PMC8990307

[ref178] Shi J. Tang J. Xu J. Jiang N. Yang Y. Chen H. . (2024). Applications of hydrogels and nanoparticles in the treatment of traumatic brain injury. Front. Bioeng. Biotechnol. 12:1515164. doi: 10.3389/fbioe.2024.1515164, 39834632 PMC11743581

[ref179] Simpson D. S. A. Oliver P. L. (2020). ROS generation in microglia: understanding oxidative stress and inflammation in neurodegenerative disease. Antioxidants (Basel). 9:743. doi: 10.3390/antiox9080743, 32823544 PMC7463655

[ref180] Siracusa L. R. Park E. Liu E. Baker A. J. (2025). Prolonged loss of nuclear HMGB1 in neurons following modeled TBI and implications for long-term genetic health. Brain Res. 1855:149559. doi: 10.1016/j.brainres.2025.149559, 40081516

[ref181] Su L. Zhang J. Gomez H. Kellum J. A. Peng Z. (2023). Mitochondria ROS and mitophagy in acute kidney injury. Autophagy 19, 401–414. doi: 10.1080/15548627.2022.2084862, 35678504 PMC9851232

[ref182] Sun J. Liu J. Gao C. Zheng J. Zhang J. Ding Y. . (2022). Targeted delivery of PARP inhibitors to neuronal mitochondria via biomimetic engineered nanosystems in a mouse model of traumatic brain injury. Acta Biomater. 140, 573–585. doi: 10.1016/j.actbio.2021.12.023, 34958970

[ref183] Sun Z. Nyanzu M. Yang S. Zhu X. Wang K. Ru J. . (2020). VX765 attenuates pyroptosis and HMGB1/TLR4/NF-kappaB pathways to improve functional outcomes in TBI mice. Oxidative Med. Cell. Longev. 2020:7879629. doi: 10.1155/2020/7879629 PMC7181015 32377306

[ref184] Sun N. Victor M. B. Park Y. P. Xiong X. Scannail A. N. Leary N. . (2023). Human microglial state dynamics in Alzheimer's disease progression. Cell 186, 4386–4403 e4329. doi: 10.1016/j.cell.2023.08.037 37774678 PMC10644954

[ref185] Surico P. L. Barone V. Singh R. B. Coassin M. Blanco T. Dohlman T. H. . (2025). Potential applications of mesenchymal stem cells in ocular surface immune-mediated disorders. Surv. Ophthalmol. 70, 467–479. doi: 10.1016/j.survophthal.2024.07.008, 39097173

[ref186] Tabassum S. Wu S. Lee C. H. Yang B. S. K. Gusdon A. M. Choi H. A. . (2025). Mitochondrial-targeted therapies in traumatic brain injury: from bench to bedside. Neurotherapeutics 22:e00515. doi: 10.1016/j.neurot.2024.e00515, 39721917 PMC11840356

[ref187] Tabet M. El-Kurdi M. Haidar M. A. Nasrallah L. Reslan M. A. Shear D. . (2022). Mitoquinone supplementation alleviates oxidative stress and pathologic outcomes following repetitive mild traumatic brain injury at a chronic time point. Exp. Neurol. 351:113987. doi: 10.1016/j.expneurol.2022.113987 35065054

[ref188] Taheri S. Karaca Z. Mehmetbeyoglu E. Hamurcu Z. Yilmaz Z. Dal F. . (2022). The role of apoptosis and autophagy in the hypothalamic-pituitary-adrenal (HPA) axis after traumatic brain injury (TBI). Int. J. Mol. Sci. 23:15699. doi: 10.3390/ijms232415699, 36555341 PMC9778890

[ref189] Tan F. Li X. Wang Z. Li J. Shahzad K. Zheng J. (2024). Clinical applications of stem cell-derived exosomes. Signal Transduct. Target. Ther. 9:17. doi: 10.1038/s41392-023-01704-0, 38212307 PMC10784577

[ref190] Tengesdal I. W. Dinarello C. A. Marchetti C. (2023). NLRP3 and cancer: pathogenesis and therapeutic opportunities. Pharmacol. Ther. 251:108545. doi: 10.1016/j.pharmthera.2023.108545, 37866732 PMC10710902

[ref191] Thapak P. Gomez-Pinilla F. (2024). The bioenergetics of traumatic brain injury and its long-term impact for brain plasticity and function. Pharmacol. Res. 208:107389. doi: 10.1016/j.phrs.2024.107389, 39243913

[ref192] Tian J. Mao Y. Liu D. Li T. Wang Y. Zhu C. (2025). Mitophagy in brain injuries: mechanisms, roles, and therapeutic potential. Mol. Neurobiol. 62, 10856–10868. doi: 10.1007/s12035-025-04936-z, 40237948 PMC12289786

[ref193] Tian J. H. Wu Q. He Y. X. Shen Q. Y. Rekep M. Zhang G. P. . (2021). Zonisamide, an antiepileptic drug, alleviates diabetic cardiomyopathy by inhibiting endoplasmic reticulum stress. Acta Pharmacol. Sin. 42, 393–403. doi: 10.1038/s41401-020-0461-z, 32647341 PMC8026994

[ref194] Tremblay-Mercier J. Madjar C. Das S. Pichet Binette A. SOM D. Étienne P. . (2021). Open science datasets from PREVENT-AD, a longitudinal cohort of pre-symptomatic Alzheimer's disease. Neuroimage Clin. 31:102733. doi: 10.1016/j.nicl.2021.102733, 34192666 PMC8254111

[ref195] Trushina E. Trushin S. Hasan M. F. (2022). Mitochondrial complex I as a therapeutic target for Alzheimer's disease. Acta Pharm. Sin. B 12, 483–495. doi: 10.1016/j.apsb.2021.11.003, 35256930 PMC8897152

[ref196] Uparela-Reyes M. J. Villegas-Trujillo L. M. Cespedes J. Velasquez-Vera M. Rubiano A. M. (2024). Usefulness of artificial intelligence in traumatic brain injury: a bibliometric analysis and mini-review. World Neurosurg. 188, 83–92. doi: 10.1016/j.wneu.2024.05.065, 38759786

[ref197] Vahab S. A. V V. K. Kumar V. S. (2025). Exosome-based drug delivery systems for enhanced neurological therapeutics. Drug Deliv. Transl. Res. 15, 1121–1138. doi: 10.1007/s13346-024-01710-x, 39325272

[ref198] van der Worp H. B. Howells D. W. Sena E. S. Porritt M. J. Rewell S. O'Collins V. . (2010). Can animal models of disease reliably inform human studies? PLoS Med. 7:e1000245. doi: 10.1371/journal.pmed.1000245, 20361020 PMC2846855

[ref199] van Erp I. A. M. Michailidou I. van Essen T. A. van der Jagt M. Moojen W. Peul W. C. . (2023). Tackling neuroinflammation after traumatic brain injury: complement inhibition as a therapy for secondary injury. Neurotherapeutics 20, 284–303. doi: 10.1007/s13311-022-01306-8, 36222978 PMC10119357

[ref200] Velmurugan G. V. Vekaria H. J. Hartz A. M. S. Bauer B. Hubbard W. B. (2024). Oxidative stress alters mitochondrial homeostasis in isolated brain capillaries. Fluids Barriers CNS. 21:81. doi: 10.1186/s12987-024-00579-9, 39407313 PMC11476969

[ref201] Vilkaite G. Vogel J. Mattsson-Carlgren N. (2024). Integrating amyloid and tau imaging with proteomics and genomics in Alzheimer’s disease. Cell Rep. Med. 5:101735. doi: 10.1016/j.xcrm.2024.101735, 39293391 PMC11525023

[ref202] Visser K. Koggel M. Blaauw J. van der Horn H. J. Jacobs B. van der Naalt J. (2022). Blood-based biomarkers of inflammation in mild traumatic brain injury: a systematic review. Neurosci. Biobehav. Rev. 132, 154–168. doi: 10.1016/j.neubiorev.2021.11.036, 34826510

[ref203] Wang D. Y. Hong M. Y. Pei J. Gao Y. H. Zheng Y. Xu X. (2021). ER stress mediated-autophagy contributes to neurological dysfunction in traumatic brain injury via the ATF6 UPR signaling pathway. Mol. Med. Rep. 23:247. doi: 10.3892/mmr.2021.11886, 33537827

[ref204] Wang K. Moore A. Grayson C. Mailloux R. J. (2023). S-nitroso-glutathione (GSNO) inhibits hydrogen peroxide production by alpha-ketoglutarate dehydrogenase: an investigation into sex and diet effects. Free Radic. Biol. Med. 204, 287–300. doi: 10.1016/j.freeradbiomed.2023.05.010, 37225107

[ref205] Wang D. Shang Q. Mao J. Gao C. Wang J. Wang D. . (2023). Phosphorylation of KRT8 (keratin 8) by excessive mechanical load-activated PKN (protein kinase N) impairs autophagosome initiation and contributes to disc degeneration. Autophagy 19, 2485–2503. doi: 10.1080/15548627.2023.2186099, 36897022 PMC10392755

[ref206] Wang D. Tai P. W. L. Gao G. (2019). Adeno-associated virus vector as a platform for gene therapy delivery. Nat. Rev. Drug Discov. 18, 358–378. doi: 10.1038/s41573-019-0012-9, 30710128 PMC6927556

[ref207] Wang B. Wang Y. Zhang J. Hu C. Jiang J. Li Y. . (2023). ROS-induced lipid peroxidation modulates cell death outcome: mechanisms behind apoptosis, autophagy, and ferroptosis. Arch. Toxicol. 97, 1439–1451. doi: 10.1007/s00204-023-03476-6, 37127681

[ref208] Wang J. Wu Z. Zhu M. Zhao Y. Xie J. (2024). ROS induced pyroptosis in inflammatory disease and cancer. Front. Immunol. 15:1378990. doi: 10.3389/fimmu.2024.1378990, 39011036 PMC11246884

[ref209] Wang Y. Zhao X. Liu-Bryan R. (2020). Role of TLR2 and TLR4 in regulation of articular chondrocyte homeostasis. Osteoarthr. Cartil. 28, 669–674. doi: 10.1016/j.joca.2020.01.011, 32007503 PMC7214200

[ref210] Wei W. Zhao Y. Zhang Y. Jin H. Shou S. (2022). The role of IL-10 in kidney disease. Int. Immunopharmacol. 108:108917. doi: 10.1016/j.intimp.2022.108917, 35729842

[ref211] Weng W. He Z. Ma Z. Huang J. Han Y. Feng Q. . (2025). Tufm lactylation regulates neuronal apoptosis by modulating mitophagy in traumatic brain injury. Cell Death Differ. 32, 530–545. doi: 10.1038/s41418-024-01408-0, 39496783 PMC11894137

[ref212] Wiseman R. L. Mesgarzadeh J. S. Hendershot L. M. (2022). Reshaping endoplasmic reticulum quality control through the unfolded protein response. Mol. Cell 82, 1477–1491. doi: 10.1016/j.molcel.2022.03.025, 35452616 PMC9038009

[ref213] Worthen R. J. Garzon Zighelboim S. S. Torres Jaramillo C. S. Beurel E. (2020). Anti-inflammatory IL-10 administration rescues depression-associated learning and memory deficits in mice. J. Neuroinflammation 17:246. doi: 10.1186/s12974-020-01922-1, 32828124 PMC7443292

[ref214] Wu Q. Gao C. Wang H. Zhang X. Li Q. Gu Z. . (2018). Mdivi-1 alleviates blood-brain barrier disruption and cell death in experimental traumatic brain injury by mitigating autophagy dysfunction and mitophagy activation. Int. J. Biochem. Cell Biol. 94, 44–55. doi: 10.1016/j.biocel.2017.11.007, 29174311

[ref215] Xie Y. Li X. Shi Q. le L. Wang C. Xu H. . (2024). The synergistic effect of curcumin and mitoquinol mesylate on cognitive impairment and the neuropathology of Alzheimer's disease. Brain Res. 1837:148959. doi: 10.1016/j.brainres.2024.148959, 38670478

[ref216] Xu J. Luo Y. Lu F. Xu X. Jiang C. Kang M. . (2025). Tauroursodeoxycholic acid modulates neuroinflammation via STING/NF-κB inhibition after traumatic brain injury. Int. Immunopharmacol. 165:115471. doi: 10.1016/j.intimp.2025.115471, 40915187

[ref217] Xu X. Sun B. Zhao C. (2023). Poly (ADP-ribose) polymerase 1 and parthanatos in neurological diseases: from pathogenesis to therapeutic opportunities. Neurobiol. Dis. 187:106314. doi: 10.1016/j.nbd.2023.106314, 37783233

[ref218] Yan Y. He M. Zhao L. Wu H. Zhao Y. Han L. . (2022). A novel HIF-2alpha targeted inhibitor suppresses hypoxia-induced breast cancer stemness via SOD2-mtROS-PDI/GPR78-UPR(ER) axis. Cell Death Differ. 29, 1769–1789. doi: 10.1038/s41418-022-00963-8 35301432 PMC9433403

[ref219] Yang Y. Lu D. Wang M. Liu G. Feng Y. Ren Y. . (2024). Endoplasmic reticulum stress and the unfolded protein response: emerging regulators in progression of traumatic brain injury. Cell Death Dis. 15:156. doi: 10.1038/s41419-024-06515-x, 38378666 PMC10879178

[ref220] Yang J. F. Xing X. Luo L. Zhou X. W. Feng J. X. Huang K. B. . (2023). Mitochondria-ER contact mediated by MFN2-SERCA2 interaction supports CD8^+^ T cell metabolic fitness and function in tumors. Sci Immunol. 8:eabq2424. doi: 10.1126/sciimmunol.abq2424, 37738362

[ref221] Yang M. Zhang Q. Luo S. Han Y. Zhao H. Jiang N. . (2023). DsbA-L alleviates tubular injury in diabetic nephropathy by activating mitophagy through maintenance of MAM integrity. Clin. Sci. (Lond.) 137, 931–945. doi: 10.1042/CS20220787, 37226722

[ref222] Yang J. Zhang X. Wang P. Guo Y. Sun K. Wu Q. . (2024). MAC: Maximal cliques for 3D registration. IEEE Trans. Pattern Anal. Mach. Intell. 46, 10645–10662. doi: 10.1109/TPAMI.2024.3442911, 39137079

[ref223] York A. G. Skadow M. H. Oh J. Qu R. Zhou Q. D. Hsieh W. Y. . (2024). IL-10 constrains sphingolipid metabolism to limit inflammation. Nature 627, 628–635. doi: 10.1038/s41586-024-07098-5, 38383790 PMC10954550

[ref224] You W. Knoops K. Berendschot T. Berendschot T. T. J. M. Benedikter B. J. Webers C. A. B. . (2024). PGC-1a mediated mitochondrial biogenesis promotes recovery and survival of neuronal cells from cellular degeneration. Cell Death Discov. 10:180. doi: 10.1038/s41420-024-01953-0, 38632223 PMC11024166

[ref225] Yu T. Hou D. Zhao J. Lu X. Greentree W. K. Zhao Q. . (2024). NLRP3 Cys126 palmitoylation by ZDHHC7 promotes inflammasome activation. Cell Rep. 43:114070. doi: 10.1016/j.celrep.2024.114070, 38583156 PMC11130711

[ref226] Zarini-Gakiye E. Vaezi G. Parivar K. Sanadgol N. (2021). Age and dose-dependent effects of alpha-lipoic acid on human microtubule-associated protein tau-induced endoplasmic reticulum unfolded protein response: implications for Alzheimer’s disease. CNS Neurol. Disord. Drug Targets 20, 451–464. doi: 10.2174/1871527320666210126114442, 33573583

[ref227] Zeng Z. Zhang Y. Jiang W. He L. Qu H. (2020). Modulation of autophagy in traumatic brain injury. J. Cell. Physiol. 235, 1973–1985. doi: 10.1002/jcp.29173, 31512236

[ref228] Zhang X. Liu H. Shi Y. Liu Z. Wang Y. Chen S. . (2025). OPTN ameliorates chondrocyte apoptosis in temporomandibular joint osteoarthritis by modulating ER-mitochondria Ca^2+^ transfer. Int. Immunopharmacol. 157:114796. doi: 10.1016/j.intimp.2025.114796, 40339496

[ref229] Zhang Y. P. Yang Q. Li Y. A. Yu M. H. He G. W. Zhu Y. X. . (2023). Inhibition of the activating transcription factor 6 branch of endoplasmic reticulum stress ameliorates brain injury after deep hypothermic circulatory arrest. J. Clin. Med. 12:814. doi: 10.3390/jcm12030814, 36769462 PMC9917384

[ref230] Zhang Z. Zhou H. Gu W. Wei Y. Mou S. Wang Y. . (2024). CGI1746 targets sigma(1)R to modulate ferroptosis through mitochondria-associated membranes. Nat. Chem. Biol. 20, 699–709. doi: 10.1038/s41589-023-01512-1 38212578

[ref231] Zhao N. Quicksall Z. Asmann Y. W. Ren Y. (2022). Network approaches for omics studies of neurodegenerative diseases. Front. Genet. 13:984338. doi: 10.3389/fgene.2022.984338, 36186441 PMC9523597

[ref232] Zhao H. Richardson C. Marriott I. Yang I. H. Yan S. (2024). APE1 is a master regulator of the ATR−/ATM-mediated DNA damage response. DNA Repair (Amst) 144:103776. doi: 10.1016/j.dnarep.2024.103776, 39461278 PMC11611674

[ref233] Zhao W. B. Sheng R. (2025). The correlation between mitochondria-associated endoplasmic reticulum membranes (MAMs) and ca(2+) transport in the pathogenesis of diseases. Acta Pharmacol. Sin. 46, 271–291. doi: 10.1038/s41401-024-01359-9, 39117969 PMC11756407

[ref234] Zhao S. Wang S. Cao L. Zeng H. Lin S. Lin Z. . (2023). Acupuncture promotes nerve repair through the benign regulation of mTOR-mediated neuronal autophagy in traumatic brain injury rats. CNS Neurosci. Ther. 29, 458–470. doi: 10.1111/cns.14018, 36422883 PMC9804054

[ref235] Zhou Y. Glass C. K. (2025). Microglia networks within the tapestry of alzheimer's disease through spatial transcriptomics. Mol. Neurodegener. 20:102. doi: 10.1186/s13024-025-00897-y, 41024223 PMC12482212

[ref236] Zhuang H. (2025). How genomics and multi-modal AI are reshaping precision medicine. Front Med (Lausanne). 12:1660889. doi: 10.3389/fmed.2025.1660889, 40933569 PMC12417403

[ref237] Zielinski C. E. (2023). T helper cell subsets: diversification of the field. Eur. J. Immunol. 53:e2250218. doi: 10.1002/eji.202250218, 36792132

[ref238] Zuin M. Cherubini A. Volpato S. Ferrucci L. Zuliani G. (2022). Acetyl-cholinesterase-inhibitors slow cognitive decline and decrease overall mortality in older patients with dementia. Sci. Rep. 12:12214. doi: 10.1038/s41598-022-16476-w, 35842477 PMC9288483

[ref239] Zyryanova A. F. Kashiwagi K. Rato C. Harding H. P. Crespillo-Casado A. Perera L. A. . (2021). ISRIB blunts the integrated stress response by allosterically antagonising the inhibitory effect of phosphorylated eIF2 on eIF2B. Mol. Cell 81, 88–103.e106. doi: 10.1016/j.molcel.2020.10.031 33220178 PMC7837216

